# Soil diazotrophs sustain nitrogen fixation under high nitrogen enrichment *via* adjustment of community composition

**DOI:** 10.1128/msystems.00547-24

**Published:** 2024-09-10

**Authors:** Mianhai Zheng, Meichen Xu, Jing Zhang, Zhanfeng Liu, Jiangming Mo

**Affiliations:** 1 Key Laboratory of Vegetation Restoration and Management of Degraded Ecosystems, Guangdong Provincial Key Laboratory of Applied Botany, South China Botanical Garden, Chinese Academy of Sciences, Guangzhou, China; 2 South China National Botanical Garden, Guangzhou, China; 3 Key Laboratory of National Forestry and Grassland Administration on Plant Conservation and Utilization in Southern China, Guangzhou, China; 4 College of Resource and Environment, University of Chinese Academy of Sciences, Beijing, China; E O Lawrence Berkeley National Laboratory, Berkeley, California, USA

**Keywords:** biological nitrogen fixation, forest soil, nitrogen addition, nitrogen deposition, nitrogen load, diazotroph community

## Abstract

Biological nitrogen (N) fixation, an important pathway of N, inputs from the atmosphere to Earth’s ecosystems, is well demonstrated to decline under N input. However, it remains unclear why N fixers sustain N fixation in many forests under high atmospheric N deposition. To address this knowledge gap, we analyzed the response of the diazotroph community to low N loads (short-term and low N addition; 3-year N addition at the rates of 25–50 kg N ha^−1^ year^−1^) vs high loads (chronic and high N addition; 9-year N addition at the rate of 150 kg N ha^−1^ year^−1^) in forest soils using high-throughput sequencing. Rates of N fixation decreased under low and high N loads (by 13%–27% and 10%–12%, respectively). Richness and alpha diversity (ACE and Chao1) of the soil diazotroph community decreased under low but not high N loads. Approximately 67.1%–74.4% of the *nifH* gene sequences at the OTU level overlapped between the control and low N loads, but only 52.0%–53.6% of those overlapped between the control and high N loads, indicating a larger shift of diazotroph community composition under high N loads. Low N loads increased soil NH_4_^+^ concentrations, which decreased diazotroph community richness, diversity, and N fixation rates, whereas the increased soil NH_4_^+^ concentrations under high N loads did not have negative impacts on the structure and function of the diazotroph community. These findings indicate that diazotrophs sustain N fixation under high N deposition *via* adjustment of their community composition in forest soils.

**IMPORTANCE:**

This study examined the changes in soil diazotroph community under different loads of simulated N deposition and analyzed its relationship with N fixation rates in in five forests using high-throughput sequencing. The magnitudes of N fixation rates reduced by low N loads were higher than those by high N loads. Low N loads decreased richness and diversity of diazotroph community, whereas diazotroph community structure remained stable under high N loads. Compared with low N loads, high N loads resulted in a less similarity and overlap of *nifH* gene sequences among the treatments and a larger adjustment of diazotroph community. Low N loads increased soil NH4+ concentrations, which decreased diazotroph community richness, diversity, and N fixation rates, whereas the increased soil NH4+ under high N loads did not have negative impacts on diazotroph community structure and N fixation. Based on these findings, it is urgently needed to incorporate the loads of N deposition and the composition of diazotroph community into terrestrial N-cycling models for accurate understanding of N inputs in forest ecosystems.

## INTRODUCTION

With elevation in anthropogenic nitrogen (N) deposition worldwide (1), the fate of biological N fixation (including symbiotic and free-living N fixation) becomes uncertain (2, 3). Biological N fixation is an important process through which diazotrophs reduce atmospheric dinitrogen gas (N_2_) to biological available N (NH_3_). Nevertheless, it is estimated that the rates of anthropogenic N deposition have exceeded those of biological N fixation from continents or oceans (4, 5). Although N deposition and anthropogenic N fertilization commonly reduce the rates of biological N fixation (6–8), they do not inhibit biological N fixation entirely (9). Increasing lines of evidence show that free-living diazotrophs sustain N fixation in many forests despite high soil N concentrations (10–13). Thus, the role of free-living N fixation in forest ecosystems needs revaluation in the context of N deposition.

Currently, our understanding of the N-deposition impacts on free-living N fixation has several limitations. First, the traditional views hold that biological N fixation declines with N enrichment because the added N (e.g., NH_4_^+^) can suppress nitrogenase synthesis (14) and/or because N fixers have less competitive advantage when ambient N is not limiting (15). These views contradict the observation that high rates of free-living N fixation occur in many forests under N enrichment (10). For example, the rates of free-living N fixation remained high (~15 kg N ha^−1^ year^−1^) in a tropical rainforest in Costa Rica, although the soils were rich in N, as characterized by a high soil N availability relative to plant demand (12). Free-living diazotrophs contributed 10–12 kg ha^−1^ year^−1^ of N to a mature subtropical forest in southern China despite high soil N stocks and atmospheric N deposition (16). Finally, rates of free-living N fixation in forest soils remained stable across both long-term deposition (34–50 kg N ha^−1^ year^−1^) or fertilization (50–150 kg N ha^−1^ year^−1^ ) (16–18). These paradoxical phenomena indicate our poor understanding of the controls over free-living N fixation under N enrichment.

Second, although there have been many studies exploring abiotic factors that regulate free-living N fixation, studies focused on biotic controls are rare. For example, N addition can have contrasting impacts on the rates of free-living N fixation, with previous studies observing no (17), negative (19–21), or positive (22) effects. The direction and magnitude of N fixation variability are affected by resource availability (16), doses of treatments (23), ecosystem types (6), and climatic conditions (7). However, these abiotic factors have limited power to explain the variability in N fixation rates under different N-addition scenarios (6, 8). Our knowledge of the biotic factors (e.g., structure and composition of diazotroph community) regulating free-living N fixation remains sparse, which impedes our mechanistic understanding of terrestrial N fixation under N enrichment (10, 24, 25).

Third, many N-addition experiments are either short-term (e.g., <3 years) or add only low concentrations of N relative to background N deposition rates (e.g., 5–25 kg ha^−1^ year^−1^) (17, 21, 23, 26, 27), although little is known about how high loads of N addition (considering that many regions of North America, Europe, and southeast Asia are experiencing severe anthropogenic N deposition) (1, 2) affect free-living N fixation (16). The effects from low loads of N addition may differ with those from high N loads. For example, low loads of N addition (150–200 kg N ha^−1^) have no effect on N fixation rates in forest canopy (e.g., epiphytes and canopy soils) (19, 28), although high loads of N addition (600–1,200 kg N ha^−1^) reduce the rates of canopy N fixation (18). In contrast to the changes in total rates of free-living N fixation (reduced by 17%–87%) under low loads of N treatments (50–100 kg N ha^−1^ ) (29); those under high loads of N addition (600–1,800 kg N ha^−1^) are relatively minor (reduced by 9%–26%) (13, 16). These lines of evidence indicate that the responses of diazotroph community to N addition may vary with the loads of N inputs.

**Fig 1:**
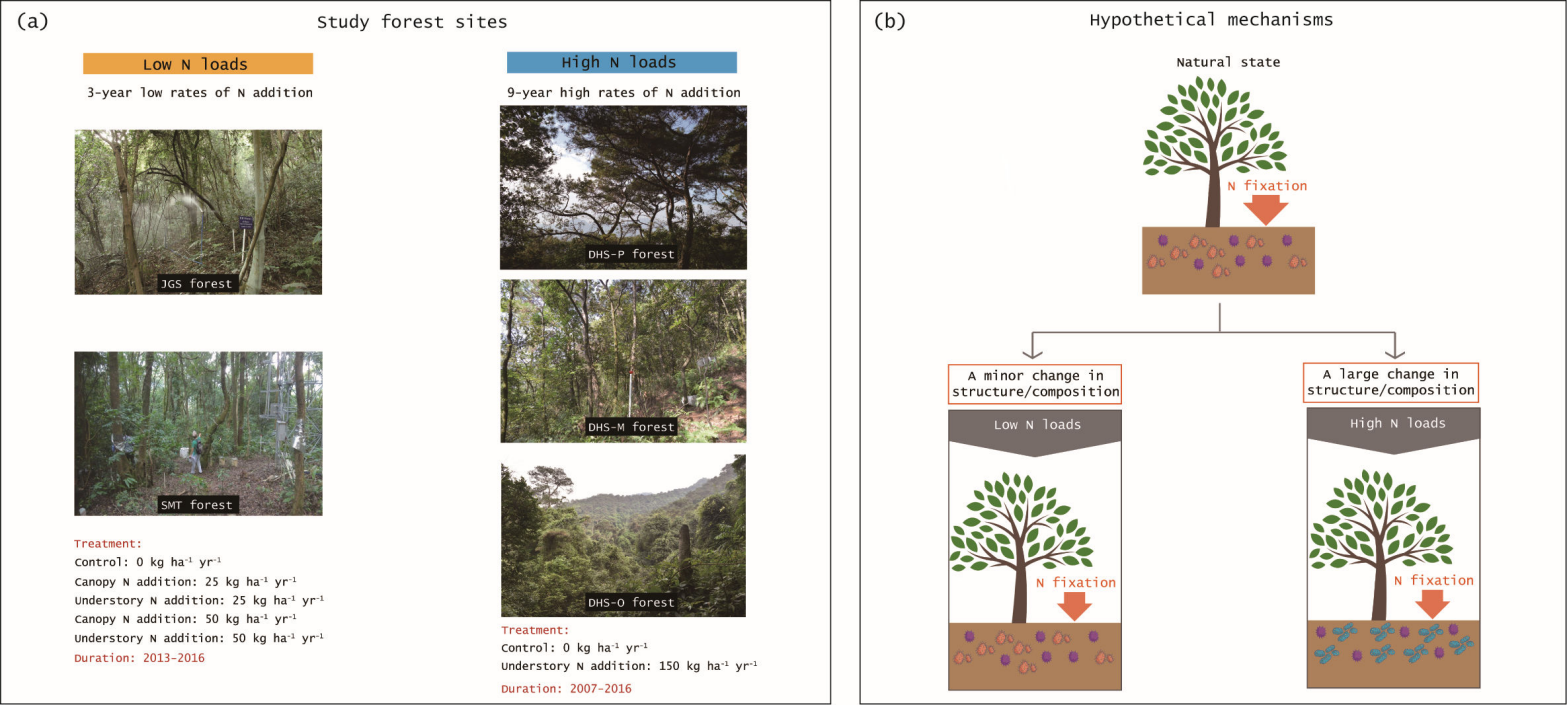
Forest sites (a) and hypothetical mechanisms (b). There are five nitrogen (**N**)-addition treatments in the Jigongshan (JGS) and Shimentai (SMT) forest sites: control (0 kg N ha^−1^ year^−1^), canopy N addition (25 and 50 kg N ha^−1^ year^−1^), and understory N addition (25 and 50 kg N ha^−1^ year^−1^) with the duration of 3 years (2013–2016). There are two N-addition treatments in the Dinghushan pine (DHS-P), mixed (DHS-M), and old-growth (DHS-O) forest sites: control (0 kg N ha^−1^ year^−1^) and understory N addition (150 kg N ha^−1^ year^−1^) with the duration of 9 years (2007–2016). We propose the hypothesis that the structure and composition of the diazotroph community have a minor change under low N loads (3-year low rates of N addition) but a large change under high N loads (9-year high rates of N addition).

To fill the knowledge gaps above, we aim to explore the response of the diazotroph community to different loads of N addition and the relationship between diazotroph community structure and N fixation rates in forests. Because free-living N fixation is pervasive in terrestrial ecosystems compared with other types (e.g., symbiotic N fixation of nodules and non-symbiotic N fixation of mosses (7, 25), our study focused on free-living diazotroph community in forest soils. We conducted an experiment of high N loads (9-year high rates of N addition) in three forests [a pine forest, a mixed forest, and an old-growth forest in Dinghushan (DHS) reserve in southern China] and another experiment of low N loads (3-year low rates of N addition) in two forests [Jigongshan (JGS) and Shimentai (SMT) forests in central and southern China, respectively; Fig. 1a]. Here, we hypothesized that (1) N fixation rates would decrease more under high N loads than under low N loads (2); structure and composition of the diazotroph community would have greater changes under high N loads than under low N loads (see hypothetical mechanisms in Fig. 1b); and (3) the N-induced changes in structure of diazotroph community would affect N fixation rates, which would vary with N loads.

## MATERIALS AND METHODS

### Site description

The low N-load experiment was conducted in the Jigongshan (JGS; 31°46′−31°52′ N, 114°01′−114°06′ E) and Shimentai (SMT; 24°22′−24°31′ N, 113°05′−113°31′ E) National Nature Reserves, which are located in Henan and Guangdong Provinces of China, respectively. In the JGS and SMT forests, the mean annual temperature (MAT) is 15.2°C and 20.8°C, and the mean annual precipitation (MAP) is 1,119 and 2,364 mm, respectively. The two forests have a similar age (45−50 years) and are dominated by broadleaved trees (*Liquidambar formosana* Hance. and *Quercus variabilis* Bl. in the JGS forest and *Castanopsis chinensis* Hance., *Schefflera octophylla* Harms., and *Schima superba* Chardn. in the SMT forest) (30). The soils are acidic with the pH of 3.5−4.4 (31).

The high N-load experiment was conducted in the Dinghushan (DHS; 23°10′ N, 112°10′ E) Biosphere Reserve located in Guangdong Province of southern China. There are three forests in this Reserve: a pine forest (DHS-P), a mixed pine and broadleaved forest (DHS-M), and an old-growth broadleaved forest (DHS-O). MAT and MAP are 21.0℃ and 1927 mm, respectively in the Reserve. The DHS-P forest (80 years old) is dominated by *Pinus* (*P*.) *massoniana*. Due to natural regeneration and invasion by native broadleaved species, the DHS-M forest (80 years old) that was originally dominated by *P*. *massoniana* is co-dominated by coniferous species (*P*. *massoniana*) and broadleaved species of *Castanopsis* (*C*.) *chinensis* and *Schima* (*S*.) *superba*. The DHS-O forest (>400 years old) experiences long-term succession and development, and it is dominated by multiple broadleaved species, including *C. chinensis*, *S. superba*, *Machilus chinensis*, and *Cryptocarya chinensis* (32). The soil pH of three forests ranges from 3.8 to 4.1 (32).

### Experimental design

#### The low N-load Experiment

In the JGS and SMT forests, N was added using two approaches: canopy and understory N addition (Fig. 1a). There are three blocks that are randomly distributed in each forest. Each block contains five N-addition treatments: control (C; 0 kg N ha^−1^ year^−1^), canopy N addition at 25 kg N ha^−1^ year^−1^ (CN25), and 50 kg N ha^−1^ year^−1^ (CN50), and understory N addition at 25 kg N ha^−1^ year^−1^ (UN25) and 50 kg N ha^−1^ year^−1^ (UN50). Each treatment was assigned to a circular plot (17 m in semi-diameter), with a total of 15 plots in each forest. Each plot was surrounded by a 20-m-wide buffer strip. From April 2013 to July 2016, NH_4_NO_3_ solutions, equivalent to 3 mm of precipitation, were applied during the growing season (from April to October) in the treatment plots (30). Treatment of canopy N addition was applied via a 35 m high spraying system, in which NH_4_NO_3_ solutions were pumped to the top of forest canopy through PVC pipes and evenly sprayed on the canopy (21). Treatment of understory N addition was applied via a 1.5-m-high irrigation system, which sprayed NH_4_NO_3_ solutions on the forest floor directly. In this experiment, the total N loads were 75−150 kg N ha^−1^.

#### The high N-load Experiment

In the DHS-P, DHS-M, and DHS-O forests, N was added on the forest floor. Two N-addition levels (each in five replicates) were designed in the DHS forests: 0 and 150 kg N ha^−1^ year^−1^. Each square plot (5 × 5 m) was surrounded by a 10 m wide buffer strip, and all the plots were randomly laid out within the forests. Solutions of NH_4_NO_3_ were sprayed on the forest floor bimonthly from February 2007 to July 2016 using a backpack sprayer. Fertilizer was mixed with 5 L of water for the N-addition plots, and the control plots were received an equivalent volume of water (13). In this experiment, the total N loads were 1350 kg N ha^−1^.

The low and high N-load experiments were set up based on the following criteria. First, we used the N-addition rates of 25−50 kg N ha^−1^ year^−1^ in the JGS and SMT forests, which are close to the background N-deposition rates of 19−34 kg N ha^−1^ year^−1^ (21). By contrast, we applied the N-addition rates of 150 kg N ha^−1^ year^−1^ in the DHS forests, approximately 3−4-folds higher than the background N deposition rates of 35−43 kg N ha^−1^ year^−1^ (13). Second, N addition enhanced leaf N contents and did not affect soil microbial biomass in the JGS or SMT forests (30, 33). By contrast, in the DHS forests, N addition did not increase leaf N contents but reduced soil microbial biomass, and meanwhile, it resulted in soil N richness and saturation with N losses via NO_3_^-^ leaching and N_2_O emission (33–35). These observations indicate that the N treatments are “high N load” in the DHS forests. In addition, despite the differences in soil nutrient status among the five forests (Table S2), we found their contribution to the variability of diazotroph community structure and N fixation following N treatments to be minor (*P* > 0.05; Fig. 7).

### Soil sampling and chemical analyses

Soil sampling was performed in July 2016. Three to five bulk soil samples were randomly collected to a depth of 10 cm in each plot using a 2.5 cm soil corer, and the samples were composited by plot evenly. A portion of the soil samples was used for measurement of chemical properties and N fixation rates (13), and another portion was stored at −80°C for analysis of diazotroph community.

Total organic C (TOC) concentrations of soils were measured by potassium dichromate oxidation titration with Fe^2+^ solution (Table S1; 35). Total N (TN) and total *P* (TP) concentrations of soils were measured by micro-Kjeldahl digestion followed by indophenol blue and Mo-Sb colorimetric methods, respectively (36). Soil ammonium (NH_4_^+^) and nitrate (NO_3_^-^) concentrations were measured by extraction in potassium chloride (KCl) solutions and analyzed using a UV-8000 spectrophotometer (37). Soil pH was measured in a soil–water (1:2.5) suspension (Table S2; (35)). N fixation rates of soils were measured using acetylene reduction assay (ARA), which measures the ability of nitrogenase to reduce acetylene to ethylene (38). N fixation rates per unit mass were expressed as the ethylene production rates (nmol C_2_H_4_ g^−1^ dry soil h^−1^) as well as the N_2_ fixation rates (ng N_2_ g^−1^ dry soil h^−1^) based on the conversion ratios measured in our forest sites (16, 21).

### High-throughput sequencing and bioinformatics analyses

Soil DNA was extracted from 0.5 g of fresh soil using a Fast DNA SPIN Kit (MP, Biomedicals, Santa Ana, CA, USA) following the manufacturer’s instructions. The *nifH* genes were amplified using the primer pairs: *nifH*-F (5′-AAAGGYGGWATCGGYAARTCCACCAC-3′) and *nifH*-R (5′-TTGTTSGCSGCRTACATSGCCATCAT-3′) (39). PCR reactions were performed in a 50-µL mixture, which contained 1 µL of DNA template (5 ng µL^−1^), 2 µL of each primer (10 µM), 25 µL 2× PCR Ex Tap (Takara, Kusatsu, Japan), and 20 µL of double-distilled water. The PCR was run at 95°C for 3 min, followed by 35 cycles of 95°C for 10 s, 55°C for 30 s, 72°C for 30 s, and extension at 72°C for 7 min. Sequence generation was performed on the Illumina HiSeq 2500 platform (San Diego, CA, USA) after purification and library construction. All paired-end raw reads were filtered using Trimmomatic (v.0.33) for high-quality clean reads, and the paired-end clean reads were merged using FLASH (v.1.2.7). Sequences were assigned to the samples, and the effective clean tags were generated after removing the chimera using UCHIME (v.4.2). The high-quality sequences were clustered into operational taxonomic units (OTUs) at 97% similarity using USEARCH (40). The sequences were further converted into amino acid sequences and assigned for OTU sequences using the reference sequences of *nifH* from FunGene and GenBank (http://fungene.cme.msu.edu/FunGene).

### Statistical analyses

Data were tested to meet the requirements of normality and homoscedasticity using Kolmogorov–Smirnov and Levene’ tests, respectively. A one-way analysis of variance (ANOVA) followed by Tukey’s HSD test was used to examine the effects of N addition on richness and alpha diversity (ACE, Chao1, Simpson, and Shannon–Winner) of diazotroph community in the JGS and SMT forests. An independent two-tailed *t* test was used to compare the difference of richness and diversity of diazotroph community between the control and N-addition treatments in the DHS forests. ANOVA and *t* test were performed using SPSS 19.0 (SPSS Inc., Chicago, IL, USA). Rarefaction curves (by which the variation in total number of sequences between samples was reduced to make the total sequence numbers uniform) for *nifH*-gene richness were performed using the “iNEXT” package in R (v.4.1.0). Heatmap analysis of sample clustering and Venn diagrams of soil *nifH* gene sequences were performed using the Packages of “pheatmap” and “venneuler” in R (v.4.1.0), respectively. Multi-sample taxonomic trees were generated using MEGAN (v.6.21.3). Many previous studies indicate that N addition regulates terrestrial N fixation rates by changing availability of soil C and nutrients (e.g., N and *P*) (6–8). Hence, we applied structural equation models (SEMs) to establish potential relationships among N-addition treatments, diazotroph community structure (richness and diversity), and N fixation rates, with soil C (total organic C) and nutrients (total N, NH_4_^+^, NO_3_^-^, and total *P*) integrated as the indirect variables. SEMs were used to examine the importance of indirect and indirect pathways of N addition on soil C, nutrients, diazotroph community structure, and N fixation rates using AMOS 21.0 (SPSS Inc.). Co-variable analysis was used to evaluate the effect sizes of different covariates to the variation in structure and function of diazotroph community and shown by partial eta squared ($\eta_{p}^{2}$) (41)). Statistical differences were recognized at *P* < 0.05.

## RESULTS AND DISCUSSION

### N fixation rates

**Fig 2:**
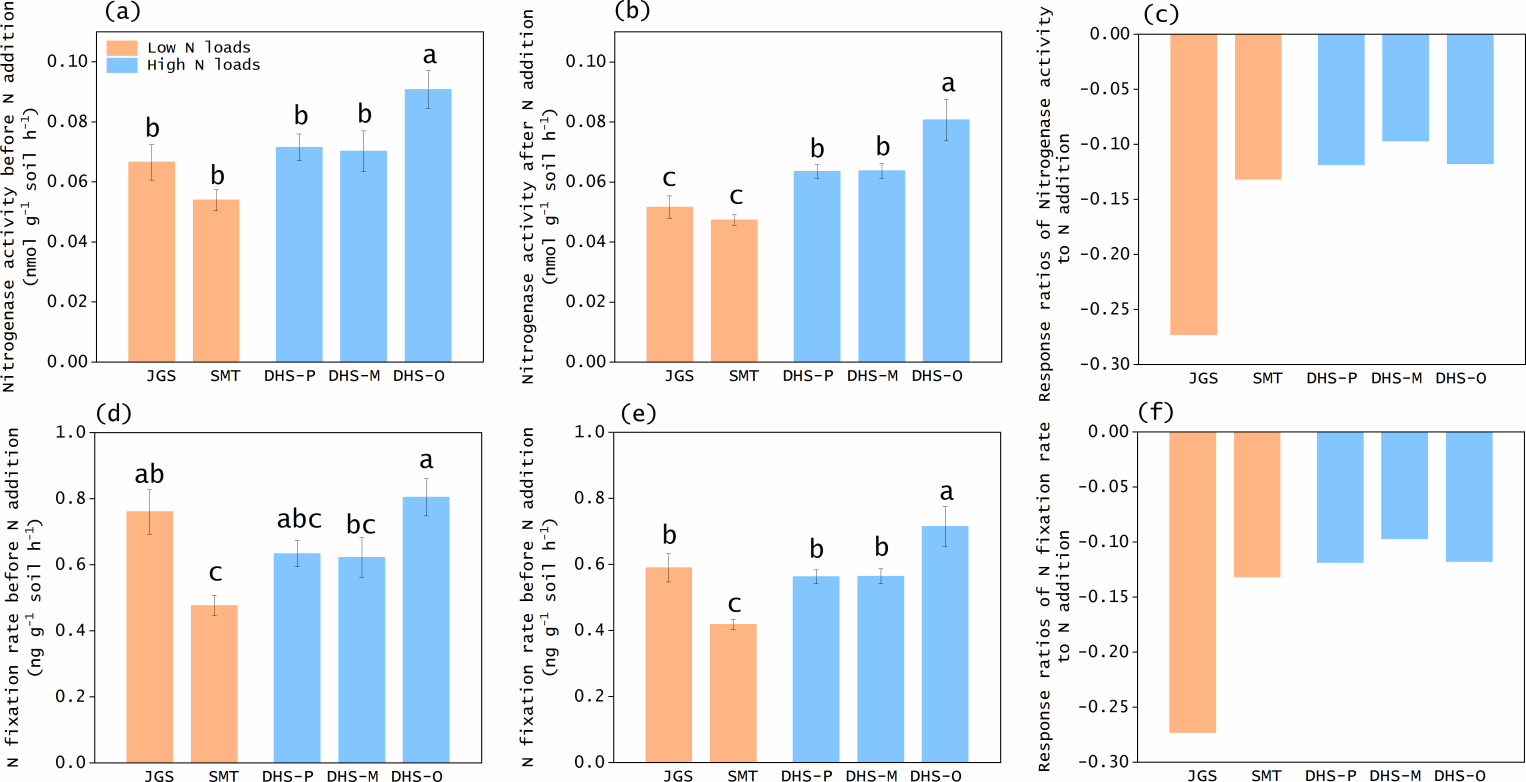
Nitrogenase activity (**a-b**) and nitrogen (**n**) fixation rate (**d-e**) before and after nitrogen (**N**) addition and their response ratios to N addition (**c, f**) in different forest soils. Error bars represent standard errors of the means. Different lowercase letters represent significant difference (*P* < 0.05) among the treatments. Shallow orange and blue colors represent low and high N loads, respectively. The same conversion ratios were applied for the control and N-addition treatments in each forest, such that the response ratios to N addition were the same between nitrogenase activity and N fixation rate. JGS: Jigongshan forest; SMT: Shimentai forest; DHS-P: Dinghushan pine forest; DHS-M: Dinghushan mixed forest; DHS-O: Dinghushan old-growth forest.

We conducted an experiment of low N loads (3 years of 25−50 kg N ha^−1^ year^−1^) in a temperate forest in JGS and a subtropical forest in SMT. Despite the difference in climate of the two forests (30), N addition decreased soil N fixation rates in both sites (*P* < 0.001 and *P* = 0.034 for JGS and SMT, respectively; Fig. 2; (21)), consistent with previous findings (19, 27, 28). Furthermore, increases in N-addition rates or duration usually lead to strongly negative impacts on N fixation rates (21, 42, 43), and we assumed a stronger impact on soil N fixation from high N loads than from low N loads.

We conducted an experiment of high N loads (9 years of 150 kg N ha^−1^ year^−1^) in three DHS forests. The 9-year N treatments generated a total N load of 1350 kg N ha^−1^, 9−18 folds as those of 3 year N treatments, but soil N fixation only had minor responses. The effect sizes under high N loads (−0.12 to −0.10) were smaller than those under low N loads (−0.27 to −0.13; Fig. 2c and f). Soil N fixation rates after high N loads (0.06−0.08 nmol g^−1^ h^−1^) were higher than those after low N loads (~0.05 nmol g^−1^ h^−1^, *P* < 0.001; Fig. 2b). These results indicate a lower sensitivity of soil N fixation in response to high N loads, allowing us to reject our hypothesis (H1).

Although total N load is equivalent to N-addition rate multiplied by duration, we found the larger contribution to statistical variation from duration than from N-addition rates. Our co-variable analysis showed that among the co-variables (i.e., N-fertilization regimes, climate, and soil physicochemical properties), N-fertilization regimes (N-addition rates, duration, and N-addition methods) were the important factors that affected diazotroph community structure and N fixation in response to N inputs (Fig. 7). The different responses of diazotroph community structure and N fixation to N addition were mainly regulated by duration of treatments (effect sizes: 0.07−0.64; Fig. 7). Although the methods of N addition (canopy vs understory pathways) could affect the amounts of N entering (or the rates of N addition) into the soils due to canopy N retention (30), the effect sizes of N-addition methods and N-addition rates on the structure of diazotroph community and rates of N fixation were lower than those of duration (Fig. 7). Thus, our results extend the past understanding that high rates of N addition have strongly negative impacts on soil N fixation compared with low rates of N addition (19, 20, 28), and importantly, indicate that these negative effects may be diluted with time (duration).

### Structure and composition of diazotroph community

**Fig 3:**
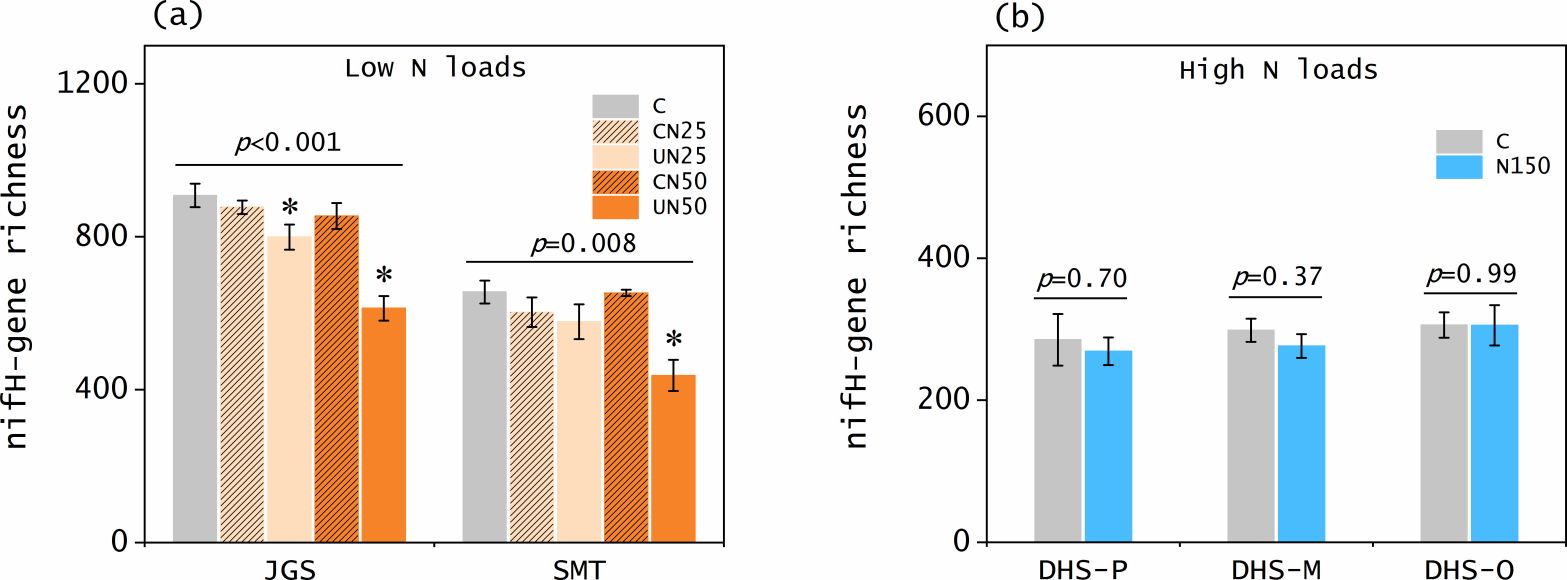
Patterns of soil *nifH*-gene richness among the treatments in five forest sites (**a-b**). Error bars represent standard errors of the means. Statistical significance is recognized at *P* < 0.05. C: control; CN25 and UN25: canopy and understory N addition at the rate of 25 kg N ha^−1^ year^−1^, respectively; CN50 and UN50: canopy and understory N addition at the rate of 50 kg N ha^−1^ year^−1^, respectively; N150: understory N addition at the rate of 150 kg N ha^−1^ year^−1^. JGS: Jigongshan forest; SMT: Shimentai forest; DHS-P: Dinghushan pine forest; DHS-M: Dinghushan mixed forest; DHS-O: Dinghushan old-growth forest.

**Fig 4:**
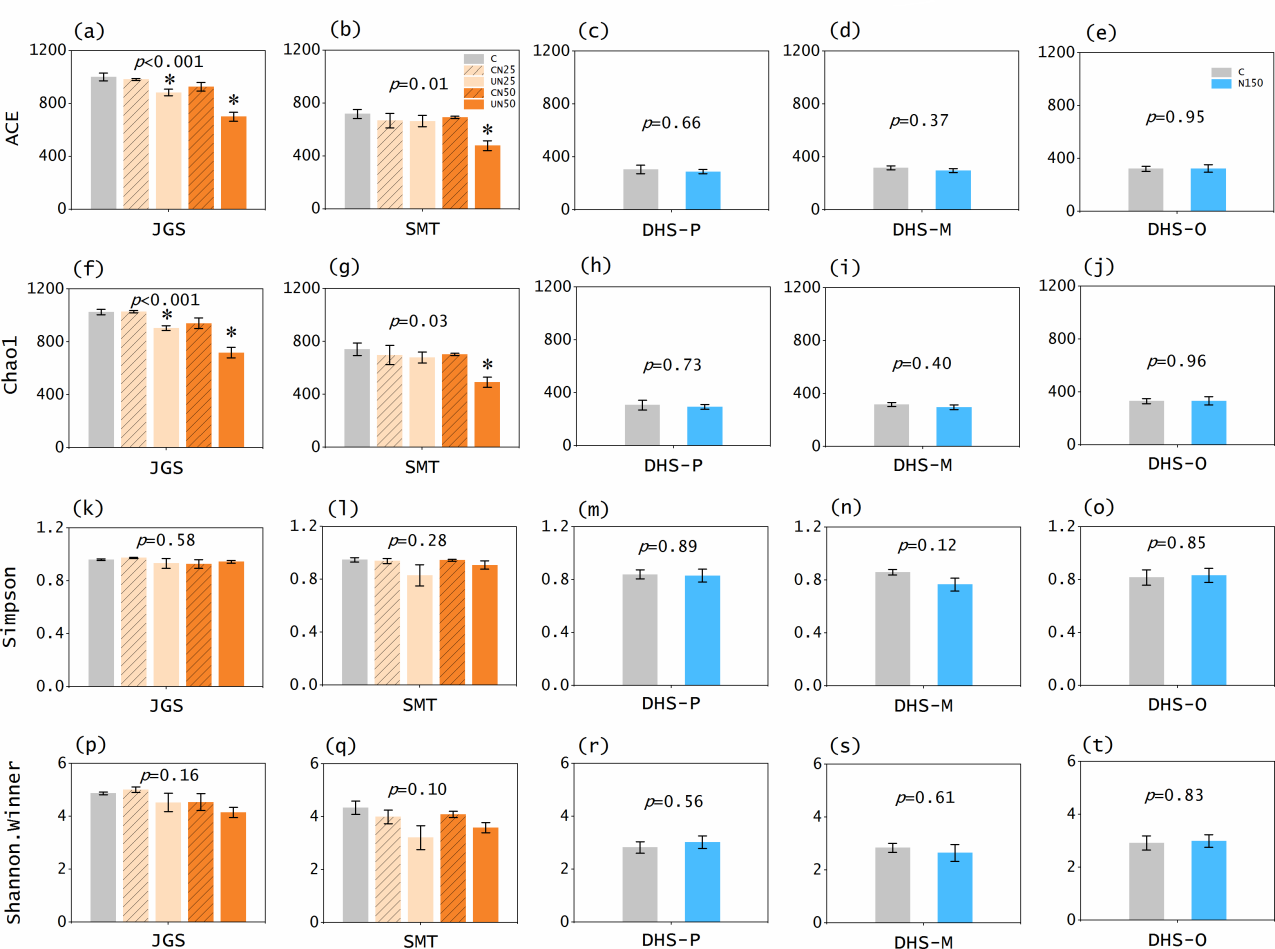
Effects of nitrogen (**N**) addition on alpha diversity [ACE (**a-e**), Chao1 (**f-j**), Simpson (**k-o**), and Shannon-Winner (**p-t**)] of diazotroph community in different forest soils. Error bars represent standard errors of the means. Statistical significance is recognized at *P* < 0.05. JGS: Jigongshan forest; SMT: Shimentai forest; DHS-P: Dinghushan pine forest; DHS-M: Dinghushan mixed forest; DHS-O: Dinghushan old-growth forest; C: control; CN25 and UN25: canopy and understory N addition at the rate of 25 kg N ha^−1^ year^−1^, respectively; CN50 and UN50: canopy and understory N addition at the rate of 50 kg N ha^−1^ year^−1^, respectively; N150: understory N addition at the rate of 150 kg N ha^−1^ year^−1^.

To explore the mechanisms underlying the N fixation responses, we identified the structure (richness and diversity) of diazotroph community. Our results showed that low N loads decreased *nifH* gene richness in the soils (by 12.0%−33.3%, *P* ≤ 0.008; Fig. 3a; Fig. S1a and b). The ACE and Chao1 of soil *nifH* genes also decreased after low N loads (by 11.7%−33.4% and 11.7%−33.5%, respectively, *P* ≤ 0.03; Fig. 4), indicating that not only the numbers but also diversity of diazotroph community decreased. These results are consistent to the patterns of N fixation rates, which were reduced by 21%−38% after N addition in the same forest soils (21). Besides our study, many recent studies also reported negative N effects on abundance, richness, and diversity of soil diazotroph community in forests (44) and farmlands (24, 45, 46).

In contrast, high N loads did not reduce soil *nifH* gene richness in any of the three DHS forests (*P* > 0.05; Fig. 3b; Fig. S1c through e). All the diversity indexes (ACE, Chao1, Simpson, and Shannon–Winner) of soil *nifH* genes were comparable between the control and N-addition plots (*P* > 0.05; Fig. 4). These results indicate that the structure of the diazotroph community was not changed by high N inputs. In certain agricultural soils, 35 years of N fertilization did not affect the copy numbers of *nifH* genes (47), which supports our findings. Compared with the variation in richness and diversity of diazotroph community under low N loads, the lack of responses under high N loads suggests that the structure of the diazotroph community was stable under high N enrichment. This supports our observation that high N loads exerted no significant effect on N fixation rates in the DHS forest soils (Fig. 2).

**Fig 5:**
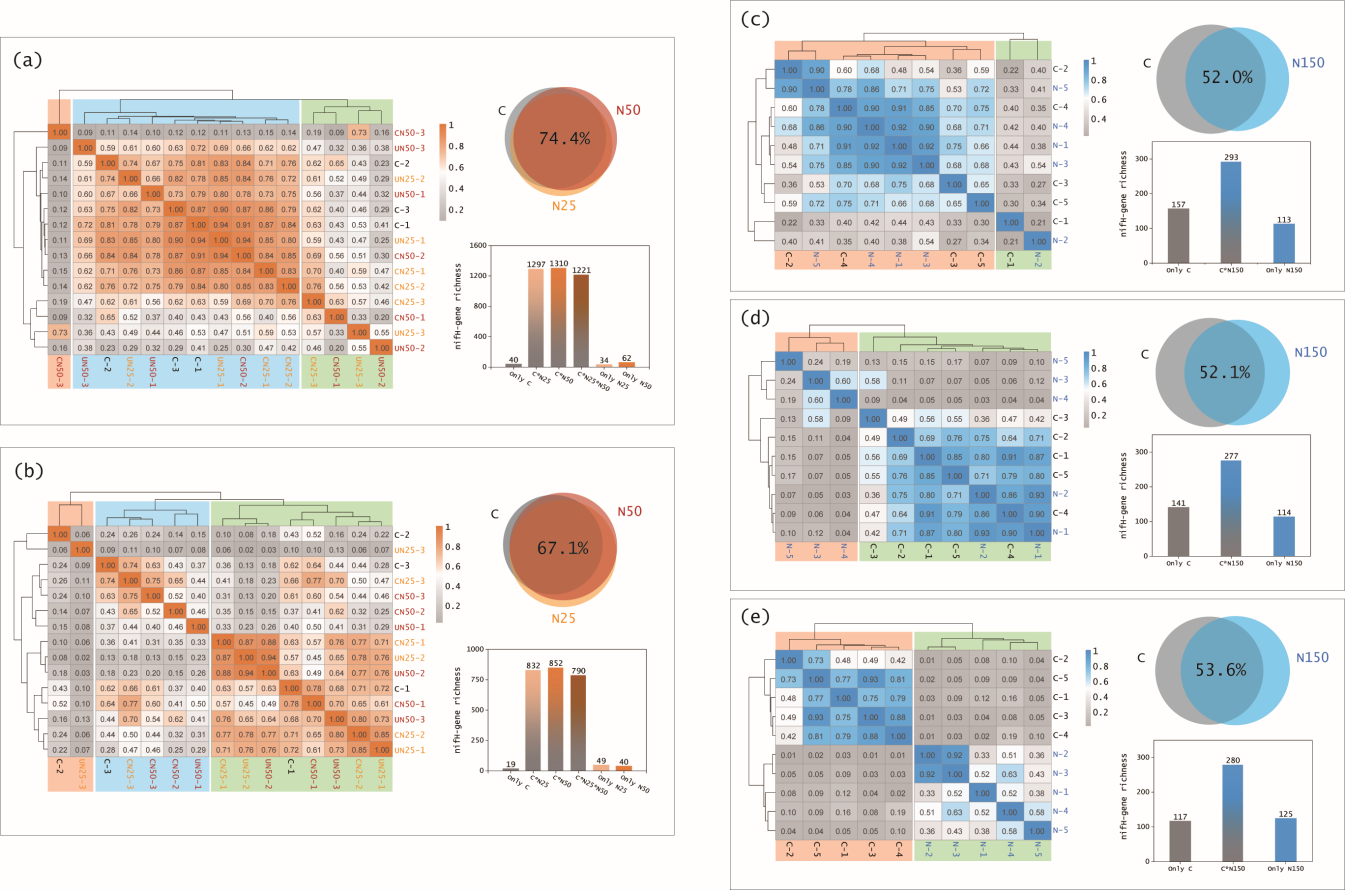
Heatmap analysis of sample cluster (left) and Venn diagrams showing the overlaps of soil *nifH* gene sequences among the treatments (right) at the OTU level. Samples are from Jigongshan (JGS) (**a**), Shimentai (SMT) (**b**), Dinghushan pine (DHS-P) (**c**), Dinghushan mixed (DHS-M) (**d**), and Dinghushan old-growth (DHS-O) (e) forest sites. Histograms show the numbers of OTUs (*nifH* gene richness) in the control plots, N-addition plots, or their overlaps. Percentage within each Venn diagram represents the overlap ratio of OUTs among the treatments. Each treatment contains three duplicated plots (samples) in the JGS and SMT forest sites or five duplicated plots (samples) in the DHS-P, DHS-M, and DHS-O forest sites. C: control; CN25 and UN25 (merged as N25): canopy and understory N addition at the rate of 25 kg N ha^−1^ year^−1^, respectively; CN50 and UN50 (merged as N50): canopy and understory N addition at the rate of 50 kg N ha^−1^ year^−1^, respectively; N150: understory N addition at the rate of 150 kg N ha^−1^ year^−1^.

Apart from the structure of diazotroph community, we also analyzed its composition (relative abundance of *nifH* genes at different taxonomic levels) based on the following two methods. First, we examined the similarity of the samples at the OTU level using clustering analysis. Our Heatmap indicated that the OTUs from control and N-addition samples grouped together under low N loads (Fig. 5a and b), but they tended to cluster separately under high N loads (Fig. 5c through e). Notably, the OTUs were clearly clustered into two contrasting groups, with one by control samples and the other by N-addition samples in the DHS-O forest (Fig. 5e). These lines of evidence suggest that high N addition altered the composition of soil diazotroph community.

Second, we examined the similarity of the samples using OTU-overlap analysis. Our Venn diagrams showed that 67.1%−74.4% of the OTUs overlapped among the treatments in the JGS and SMT forests, but only 52.0−53.6% of those overlapped in the DHS forests (Fig. 5; Fig. S2). These data suggested a large shift of diazotroph community composition under high N loads compared to those under low N loads, which partially supports our hypothesis (H2). Given the adjustment of diazotroph community composition as an important pathway by which diazotroph communities inhabit N-rich soils, it provides a new mechanism underlying the paradoxical observations that high rates of free-living N fixation occur in many mature forests despite soil N richness (10–12) and atmospheric N deposition (16).

**Fig 6:**
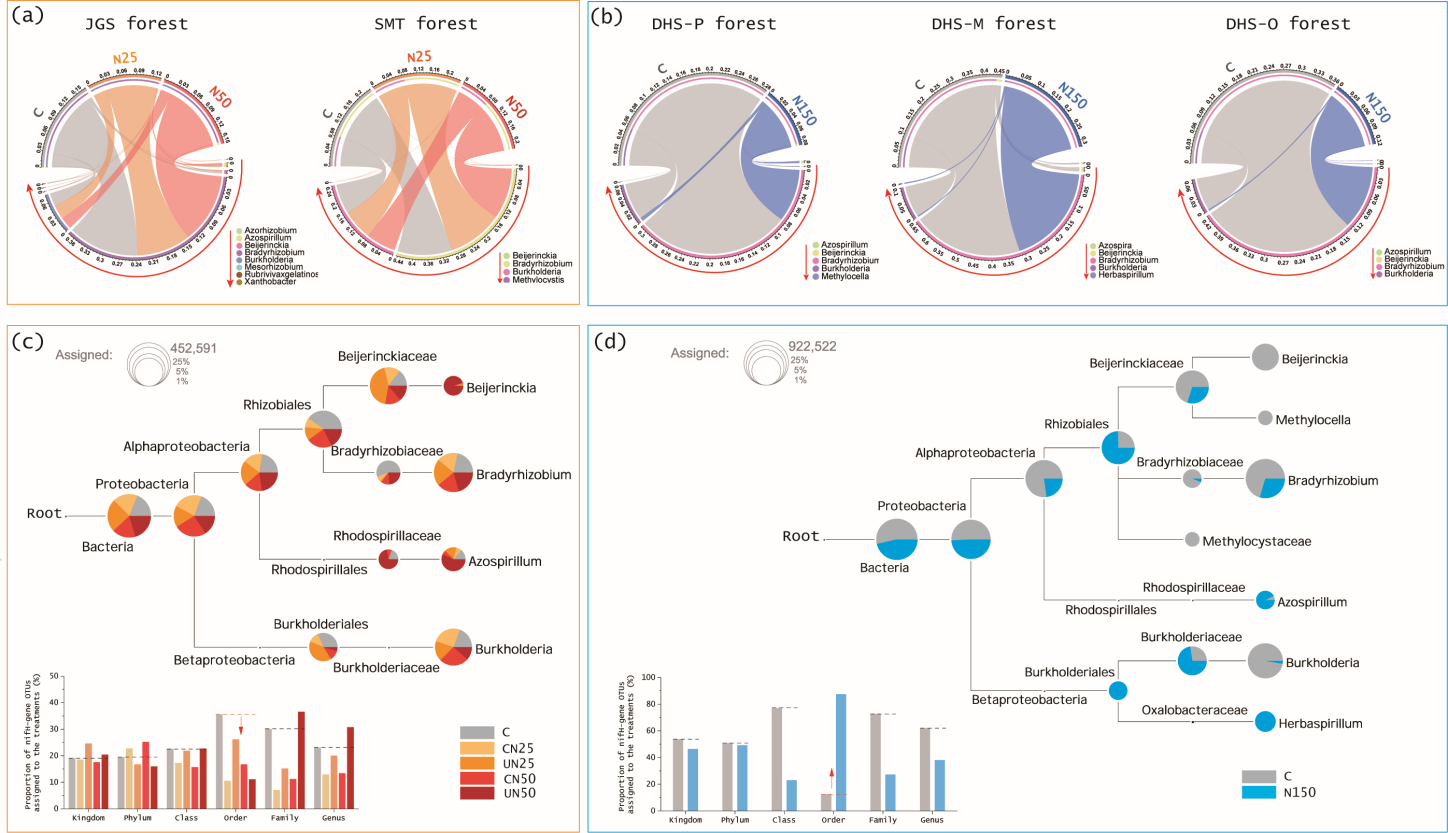
Chord diagrams of the relative abundance of soil dominant nitrogen (**N**)-fixing microbes at the genus level (**a-b**) and multi-sample taxonomic dendrograms of soil diazotrophs at the OTU level (**c-d**). The genera that cannot be identified are not shown in the chord diagrams. Area of each pie within the taxonomic trees represents the numbers of OTUs that cannot be identified into the subordinate taxon. Histograms show the proportion of OTUs that were assigned to the treatments in six branches (kingdom, phylum, class, order, family, and genus). JGS: Jigongshan forest; SMT: Shimentai forest; DHS-P: Dinghushan pine forest; DHS-M: Dinghushan mixed forest; DHS-O: Dinghushan old-growth forest; C: control; CN25 and UN25 (merged as N25): canopy and understory N addition at the rate of 25 kg N ha^−1^ year^−1^, respectively; CN50 and UN50 (merged as N50): canopy and understory N addition at the rate of 50 kg N ha^−1^ year^−1^, respectively; N150: understory N addition at the rate of 150 kg N ha^−1^ year^−1^.

In the JGS and SMT forests, the relative abundance at the genera level remained stable under low N loads (*P* > 0.05; Fig. 6a; Fig. S3a and b). In consideration of the overall declines in richness and diversity of diazotroph community in the JGS and SMT forests (Fig. 3 to 4), our richness and diversity results suggest that the abundance of diazotrophs declines under low N addition, as supported by a previous study in paddy soils (48). In contrast to forests with low N loads, the total abundance of identifiable genera was reduced in DHS forests under high loads (Fig. 6b), with significant or marginal decreases detected for *Beijerinckia* (*P* = 0.08) and *Burkholderia* (*P* = 0.03) in the DHS-M forest and for *Burkholderia* (*P* = 0.01) in the DHS-O forest (Fig. S3d and e). Because of the stability of diazotroph community richness in the DHS forests under high N loads (Fig. 3 to 4), the decreased abundance of identifiable genera (i.e., *Beijerinckia* and *Burkholderia*) accompanied with an increased trend in the abundance of non-identifiable genera (Fig. S3c through e). Thus, our findings provide the evidence that high N loads lead to adjustments of diazotroph community at the genera level, with some genera decreasing and the others tending to increase.

**Fig 7:**
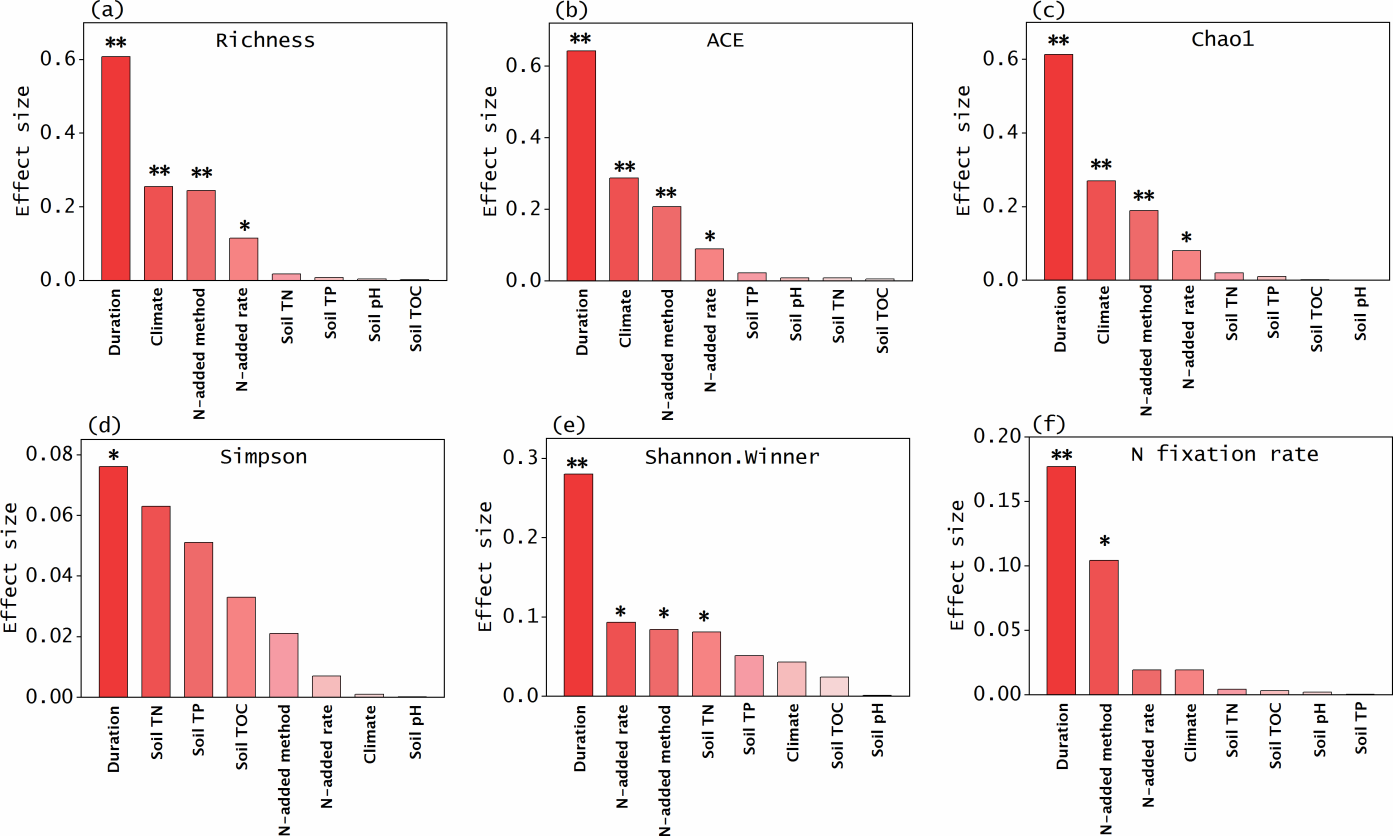
Contribution (effect sizes) of different covariates to the variation in nitrogen-(N)-fixing microbial community structure (a-e) and function (f) following N addition. Climate conditions [mean annual temperature (MAT) and precipitation (MAP)], N-added methods [canopy N addition (at 35-m height) and understory N addition (at 1.5-m height)], N-added rates (0, 25, 50, and 150 kg N ha^-1^ year^-1^), duration (3 and 9 years), soil physicochemical properties [total N (TN), total phosphorus (TP), total organic carbon (TOC), and pH] were set as covariates when the N-addition effects on diazotroph community structure and function were analyzed. Each effect size was calculated by partial eta squared ($\eta_{p}^{2}$). Due to the colinearity of MAT and MAP variables, only one of them was used for analyses. ‘*’ and ‘**’ represent statistical significance at the level of *p*<0.05 and *p*<0.01, respectively.

Furthermore, we used phylogenetic trees to validate the larger shift of diazotrophic community composition under high N loads relative to low N loads. The relative abundance of the *nifH* genes that were identifiable at multiple taxonomic levels (kingdom, phylum, class, family, and genus) decreased by 12.6% under low N loads (Fig. 6c; Fig. S4). This pattern, however, contrasts with those under high N loads, which demonstrated a 37.7% decline in relative abundance at these taxonomic levels (Fig. 7d). Specifically, the abundance of the diazotrophs that could be identified at the order level (e.g., *Rhizobiales* and *Burkholderiales*; Fig. S5) decreased by 54.7% under low N loads but increased by ~sixfolds under high N loads. These results, in combination of the changes of genera discussed above, demonstrate a large shift of diazotroph community composition under high N loads.

### Linkage of structure and function of the diazotroph community

As demonstrated above, the composition of the diazotroph community changed significantly under high N loads, with only 52.0%−53.6% of the *nifH* gene sequences showing overlap between the control and N-addition treatments. This finding is a pre-requisite for understanding of why diazotrophs can sustain N fixation under high N enrichment. Further, we link the structure (richness and diversity) of the diazotroph community to its function (N fixation rates) and hypothesized that the mechanisms regulating this coupling would be different between the low and high N loads (H3).

Under low N loads, N addition enhanced soil NH_4_^+^ concentrations, which decreased diazotroph community richness, diversity, and N fixation rates (effects sizes: 53.1%−91.6%; Fig. S6a and c). This mechanism revealed the role of soil NH_4_^+^ in regulating diazotroph community structure and function, which was supported by a prior finding that the addition of NH_4_^+^ inhibited biosynthesis of nitrogenase (14). Given that N addition commonly increases soil NH_4_^+^ concentrations, our results indicate that the elevated NH_4_^+^ concentrations caused by low N input have negative impacts on the activity of soil diazotrophs. Our findings are consistent with previous observations that low N loads (e.g., <3 years of N addition) reduces soil diazotroph biomass and N fixation rates in forests (19, 27, 29, 49), grasslands (50), croplands (51), wetlands (52), and shrublands (53).

Interestingly, high N loads also increased soil NH_4_^+^ concentrations, but the increased soil NH_4_^+^ did not decrease diazotroph community richness (*P* > 0.05), and instead, it had positive effects on diazotroph community diversity and function (effects sizes: 59.1%−81.8%; Fig. S6b and d). The positive NH_4_^+^ effects on N fixation can be supported by our previous study in a tropical forest, in which 12 years of NH_4_NO_3_ addition stimulated N fixation in the mosses and foliage by increasing photosynthetic carbohydrate products and C:N ratios in these plant tissues (16). Compared with low N addition, high N addition resulted in soil N saturation and thus N loss via NO_3_^-^ leaching or the release of N_2_O gases (33, 35), which may dilute the negative impacts of NH_4_^+^ (due to soil nitrification) on diazotroph community. In contrast to the low N-load experiment, most of the direct and indirect pathways that regulated the structure and function of diazotroph community were beneficial under high N loads (Fig. S6c, d and S7 to 10). This can, to some extent, explain why diazotrophs can freely live and sustain high N fixation rates in many N-rich tropical forests (12, 13, 16). Nevertheless, the N supply exceeds plant demand in many N-rich forests, and the soils in N-rich ecosystems commonly exhibit high loss of inorganic N (35, 54). In the future, a long-term and continuous field study incorporating N-cycling processes of soil and plant is required for a comprehensive understanding of the factors that affect the structure, composition, and N fixation of soil diazotroph community under N enrichment.

### Conclusions

In summary, our study for the first time reveals divergent mechanisms of diazotroph community regulating N fixation rates under low vs high N loads in forest soils. Rates of N fixation decreased under low and high N loads (by 13%−27% and 10%−12%, respectively). Low N loads reduced the richness and diversity of diazotroph community, whereas the diazotroph community structure remained stable under high N loads. High N loads resulted in a less similarity of *nifH* gene sequences among the treatments (i.e., 52.0%−53.6% of the *nifH* gene sequences overlapped) compared with low N loads did (67.1%−74.4%), indicating a larger shift of diazotroph community composition under high N loads. In addition, low N loads increased soil NH_4_^+^ concentrations, which decreased diazotroph community richness, diversity, and N fixation rates, whereas the increased soil NH_4_^+^ under high N loads did not have negative impacts on diazotroph community richness, diversity, and N fixation rates.

Our study has the following limitations and implications. First, the experiments of low and high N loads were performed in different forests, and thus different soil nutrient status may to some extents affect the responses of diazotroph and N cycle (55). Nevertheless, we selected multiple (2−3) forests for each experiment and found that the effects from N-addition treatments were important more than from soil nutrient status of the forest sites (Fig. 7). More studies are needed to validate our conclusions in the future. Second, our conclusions were mainly based on the changes in structure and composition of the identified diazotroph community following N treatments. Further studies are needed to examine the changes in the unidentified diazotroph community at different taxonomic levels. Despite these limitations, our findings can help understand the paradoxical phenomenon that high rates of free-living N fixation occur in already N-rich ecosystems. Furthermore, we predict that the lack of N-induced negative impacts on biological N fixation is likely to be common in forest soils if the amount of N inputs continues to increase and reach a “threshold.” Because atmospheric N deposition will likely proceed in the future (1, 5), our findings highlight the urgent needs to incorporate the composition of diazotroph community and the loads of N deposition into terrestrial N-cycling models for accurate understanding of N inputs in forest ecosystems.

## Data Availability

Data that support the findings of this study are openly available in figshare at: https://figshare.com/articles/dataset/OTU_table/25632864 and https://figshare.com/articles/dataset/N_fixation_rate/26156671.

## SUPPLEMENTAL MATERIAL

Supplemental material msystems.00547-24-s0001.doc Fig. S1-S10; Tables S1-S2.

ASM does not own the copyrights to Supplemental Material that may be linked to, or accessed through, an article. The authors have granted ASM a non-exclusive, world-wide license to publish the Supplemental Material files. Please contact the corresponding author directly for reuse.
