# Supplementary material for: Soil diazotrophs sustain nitrogen fixation under high nitrogen enrichment via adjustment of community composition
Source: mSystems. 2024 Sep 10;9(10):e00547-24. doi: 10.1128/msystems.00547-24 (PMC11495058; doi:10.1128/msystems.00547-24)
Supplement: Supplemental material — Fig. S1-S10; Tables S1-S2. [file msystems.00547-24-s0001.doc]

**Supporting Information**

**
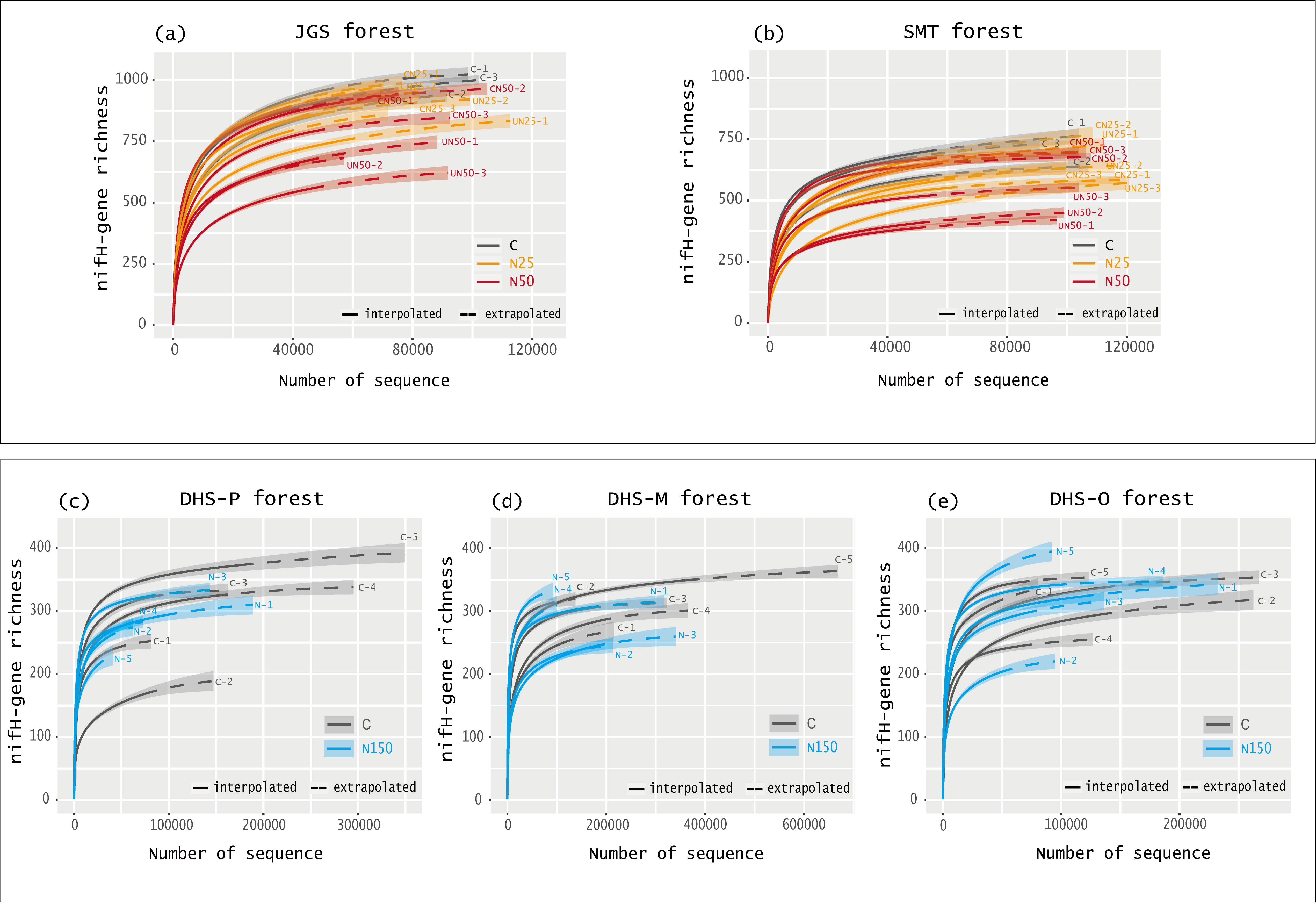
 Fig. S1** Rarefaction curves of soil *nifH*-gene richness in different nitrogen (N)-addition plots **(a-e)**. Rarefaction curves are assembled showing the numbers of OTUs, defined at 97% sequence similarity, compared to the numbers of total sequences. The solid and dashed lines of each rarefaction curve represent interpolated and extrapolated numbers of sequences that are subsampled from each sample. Shadow area represents 95% confidence interval. Each treatment contains three (labeled 1~3 at each curve) duplicated plots (samples) in the Jigongshan (JGS) and Shimentai (SMT) forest sites or five (labeled 1~5) duplicated plots (samples) in the Dinghushan pine (DHS-P), mixed (DHS-M), and old-growth (DHS-O) forest sites. C: control; CN25 and UN25 (merged as N25): canopy and understory N addition at the rate of 25 kg N ha^-1^ yr^-1^, respectively; CN50 and UN50 (merged as N50): canopy and understory N addition at the rate of 50 kg N ha^-1^ yr^-1^, respectively; N150: understory N addition at the rate of 150 kg N ha^-1^ yr^-1^.


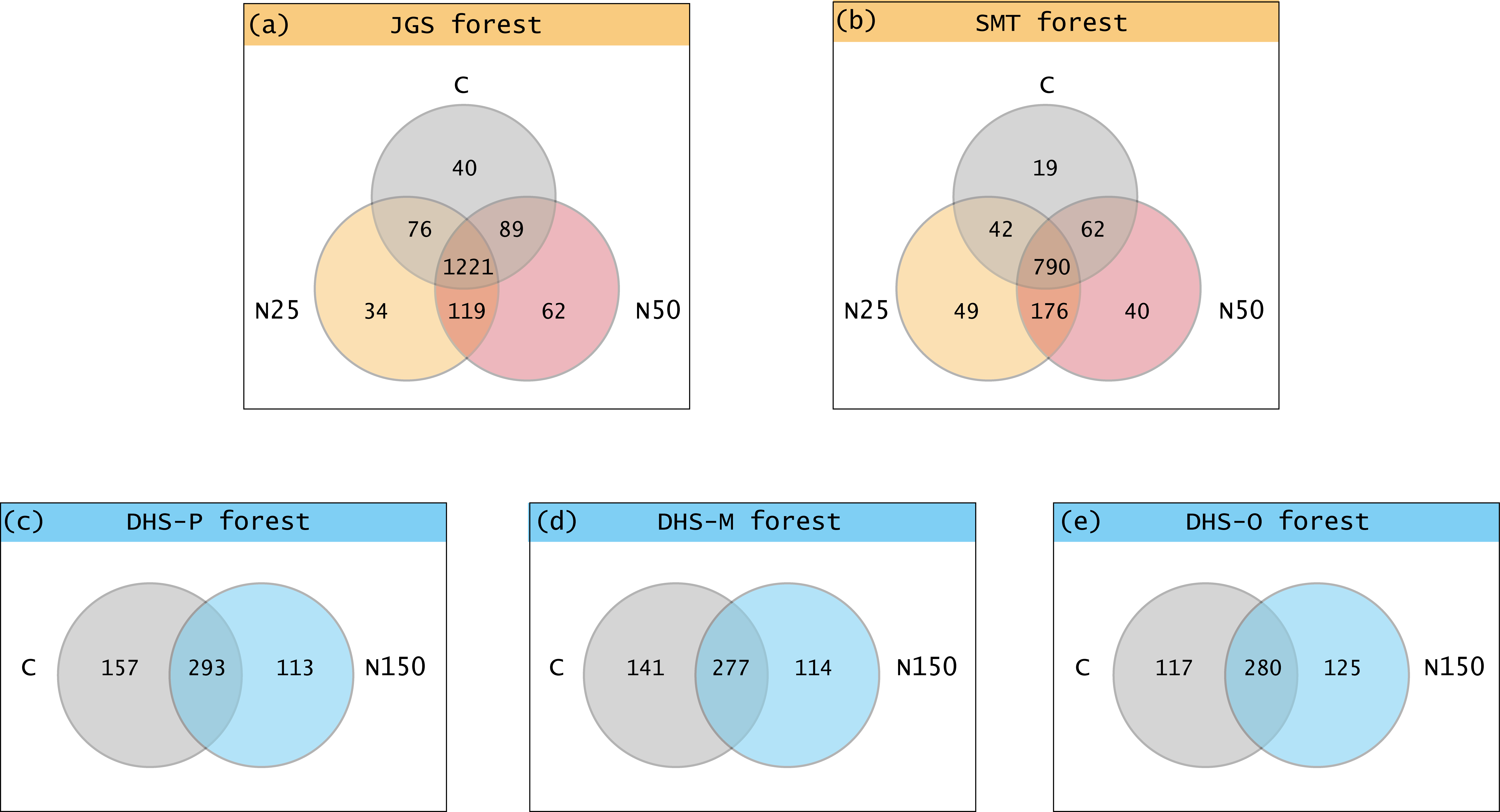


**Fig. S2** Venn diagrams showing the overlaps of soil *nifH*-gene sequences among the treatments at the OTU level. Samples are from Jigongshan (JGS) **(a)**, Shimentai (SMT) **(b)**, Dinghushan pine (DHS-P) **(c)**, Dinghushan mixed (DHS-M) **(d)**, and Dinghushan old-growth (DHS-O) **(e)** forest sites. Values on the Venn diagrams represent the numbers of OTUs. C: control; N25, N50, and N150: nitrogen addition at the rates of 25, 50, and 150 kg N ha^-1^ yr^-1^, respectively.


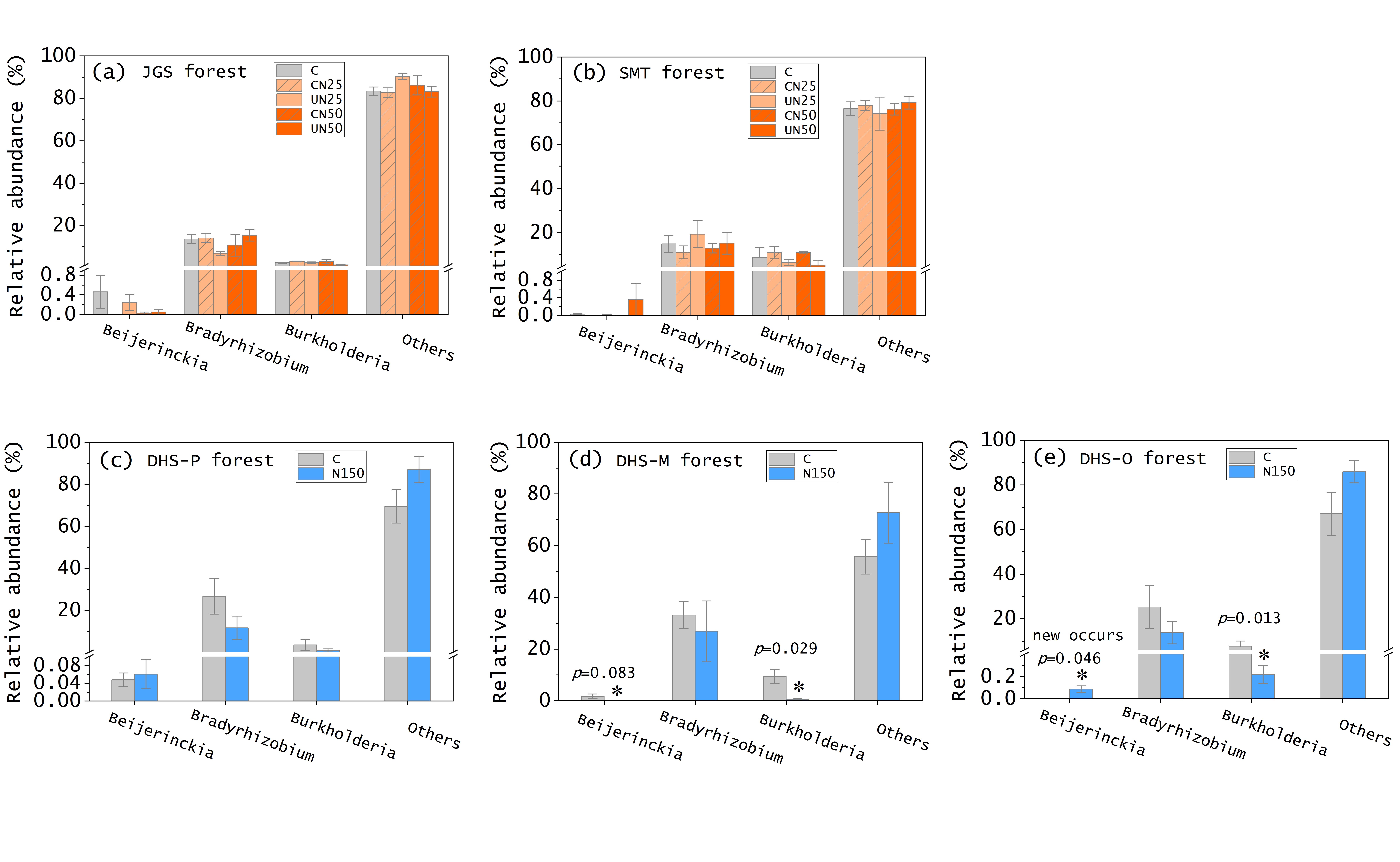


**Fig. S3** Effects of nitrogen (N) addition on the relative abundance of three dominant genera of diazotrophs in the Jigongshan (JGS) **(a)**, Shimentai (SMT) **(b)**, Dinghushan pine (DHS-P) **(c)**, Dinghushan mixed (DHS-M) **(d)**, and Dinghushan old-growth (DHS-O) **(e)** forest soils. ‘Others’ represents the combinations of the detected genera except for Beijerinckia, Bradyrhizobium, and Burkholderia. C: control; CN25 and UN25: canopy and understory N addition at the rate of 25 kg N ha^-1^ yr^-1^, respectively; CN50 and UN50: canopy and understory N addition at the rate of 50 kg N ha^-1^ yr^-1^, respectively; N150: understory N addition at the rate of 150 kg N ha^-1^ yr^-1^.


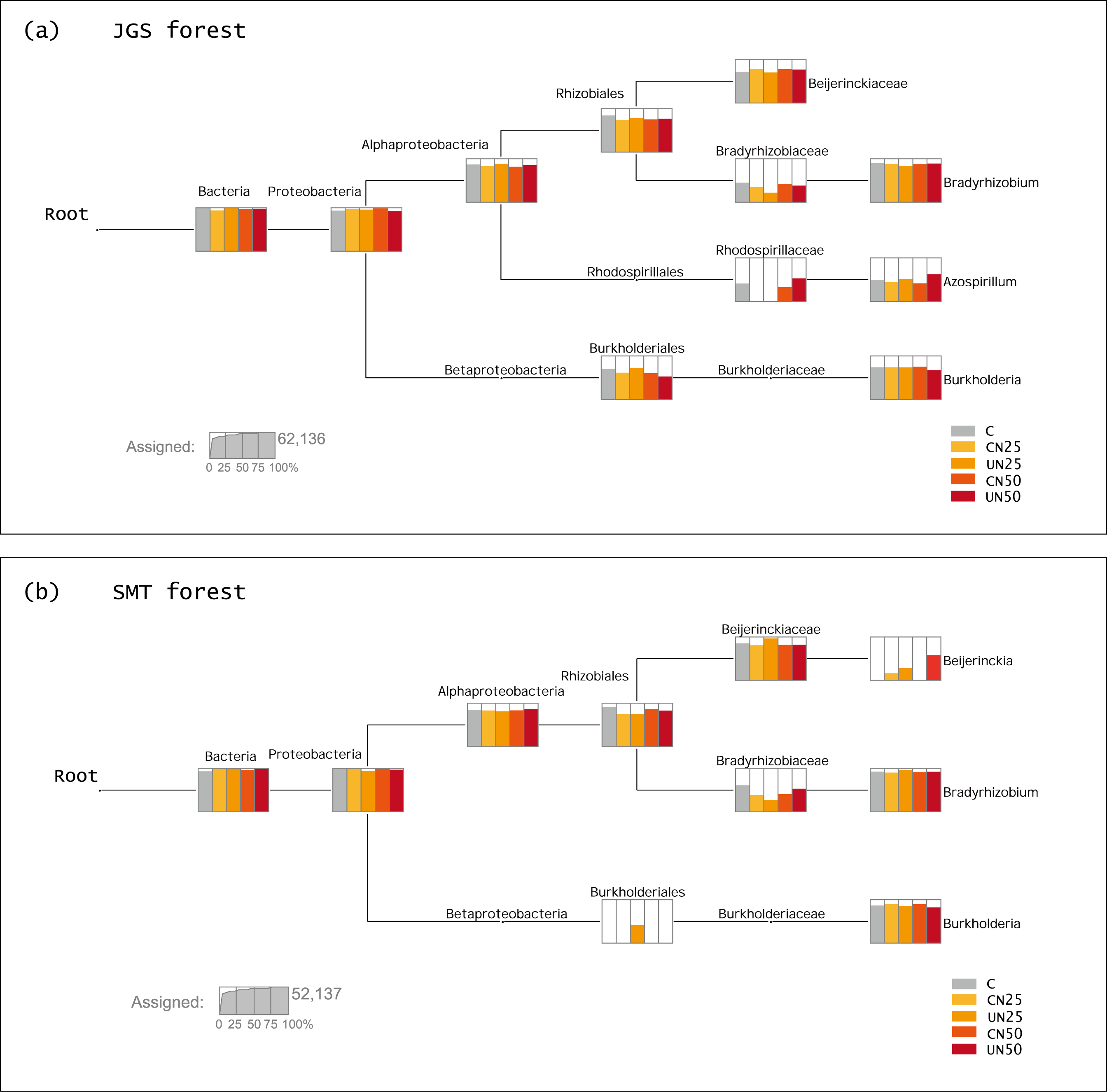


**Fig. S4** Multi-sample taxonomic dendrograms of nitrogen (N)-fixing microbes at the OUT level in the Jigongshan (JGS) **(a)** and Shimentai (SMT) **(b)** forest soils. Each column within the taxonomic trees represents the numbers of OTUs that cannot be identified into the subordinate taxon. C: control; CN25 and UN25: canopy and understory N addition at the rate of 25 kg N ha^-1^ yr^-1^, respectively; CN50 and UN50: canopy and understory N addition at the rate of 50 kg N ha^-1^ yr^-1^, respectively.


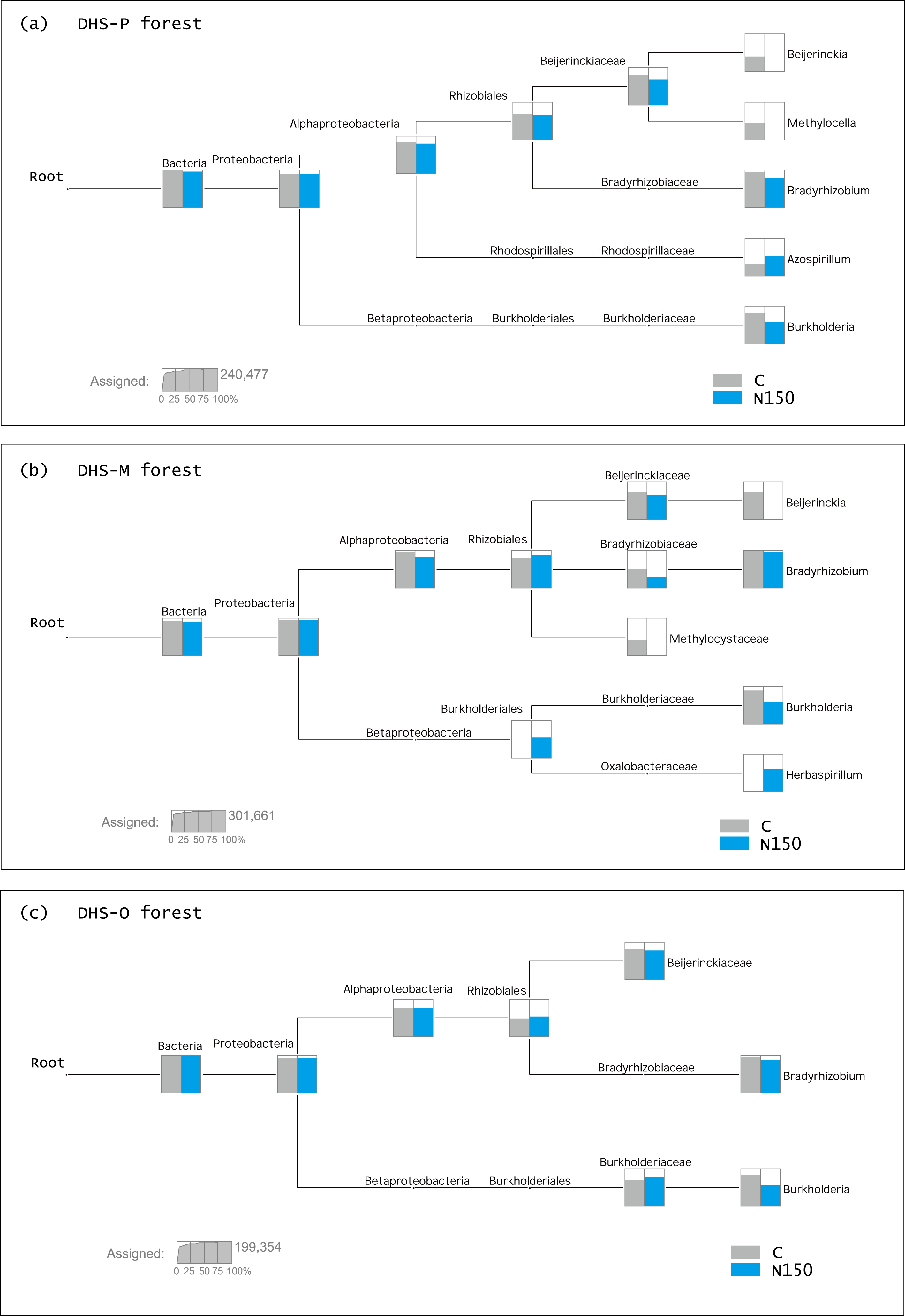


**Fig. S5** Multi-sample taxonomic dendrograms of nitrogen (N)-fixing microbes at the OUT level in the Dinghushan pine (DHS-P) **(a)**, mixed (DHS-M) **(b)**, and old-growth (DHS-O) **(c)** forest soils. Each column within the taxonomic trees represents the numbers of OTUs that cannot be identified into the subordinate taxon. C: control; N150: understory N addition at the rate of 150 kg N ha^-1^ yr^-1^.


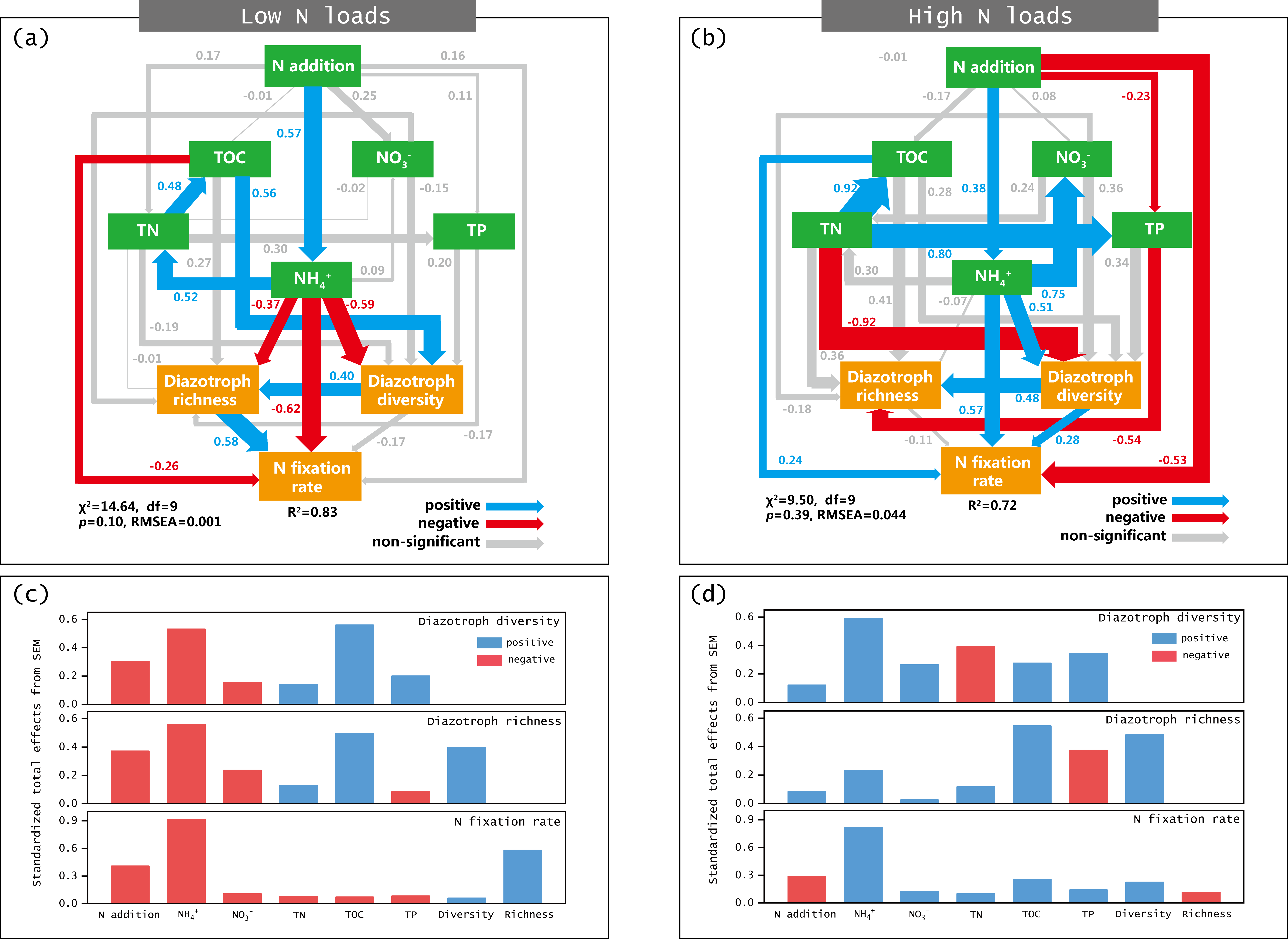


**Fig. S6** Structure equation models (SEM) showing the standardized direct, indirect, and total effects of nitrogen (N) addition, carbon (C) and nutrient concentrations on soil diazotroph richness, diversity, and N fixation rates. The left panels represent the treatment of low N loads **(a, c)**, and the right panels represent the treatment of high N loads **(b, d)**. Solid arrows represent the hypothesized direction of causation. Blue, red, and gray arrows represent positive, negative, and non-significant (*p*>0.05) relationships, respectively. Values on the arrows represent standardized path coefficients, proportional to the arrow width. R^2^ indicates the variation of N fixation rates explained by the models. TOC: total organic carbon; TN: total nitrogen; TP: total phosphorus; NO_3_^-^: nitrate; NH_4_^+^: ammonium.


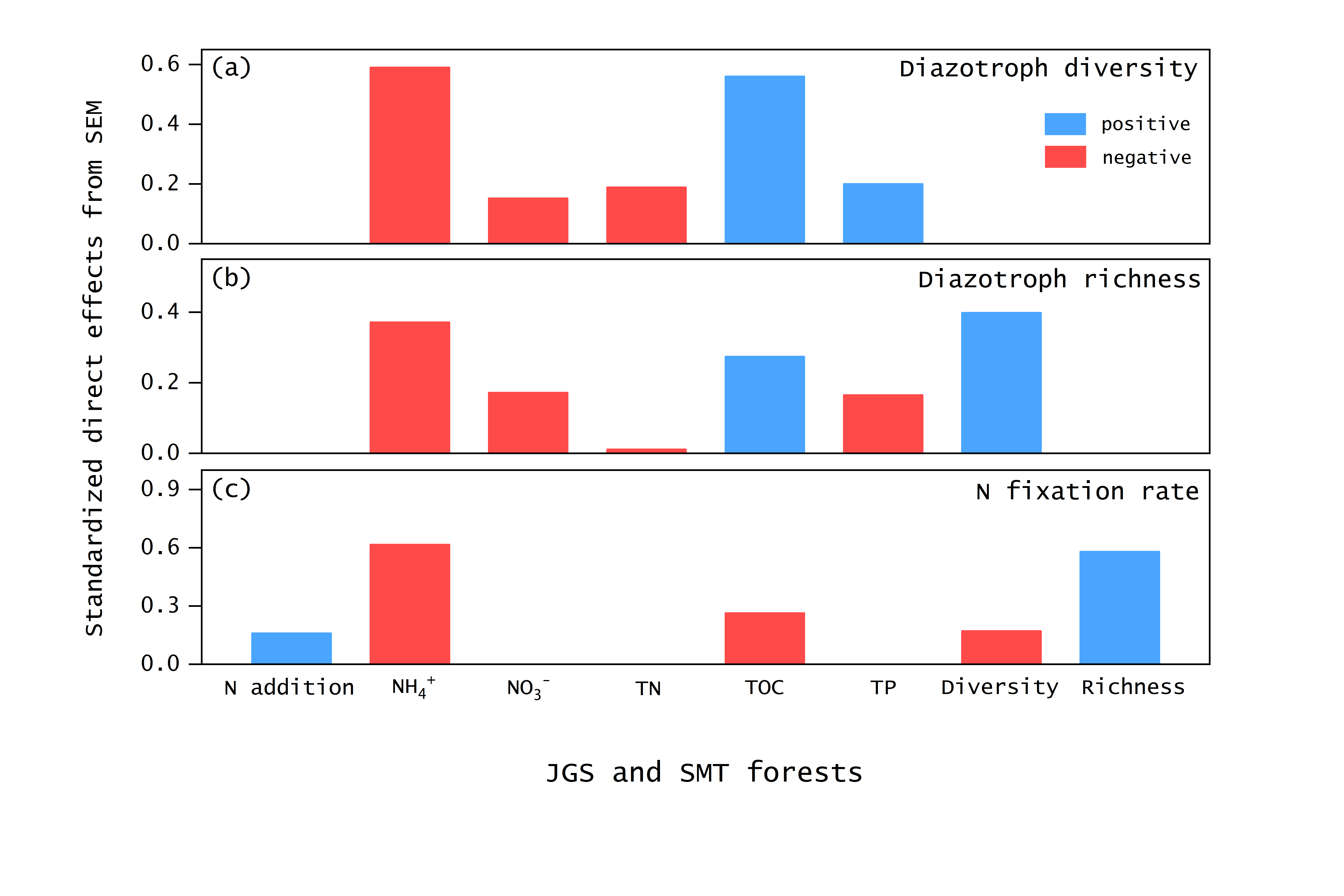


**Fig. S7** Standardized direct effects of nitrogen (N) addition, carbon (C) and nutrient concentrations on diazotroph richness **(a)**, diversity **(b)**, and N fixation rates **(c)** in the Jigongshan (JGS) and Shimentai (SMT) forest soils from structure equation models (SEM). Blue and red colors represent positive and negative effects, respectively. TOC: total organic carbon; TN: total nitrogen; TP: total phosphorus; NO_3_^-^: nitrate; NH_4_^+^: ammonium.


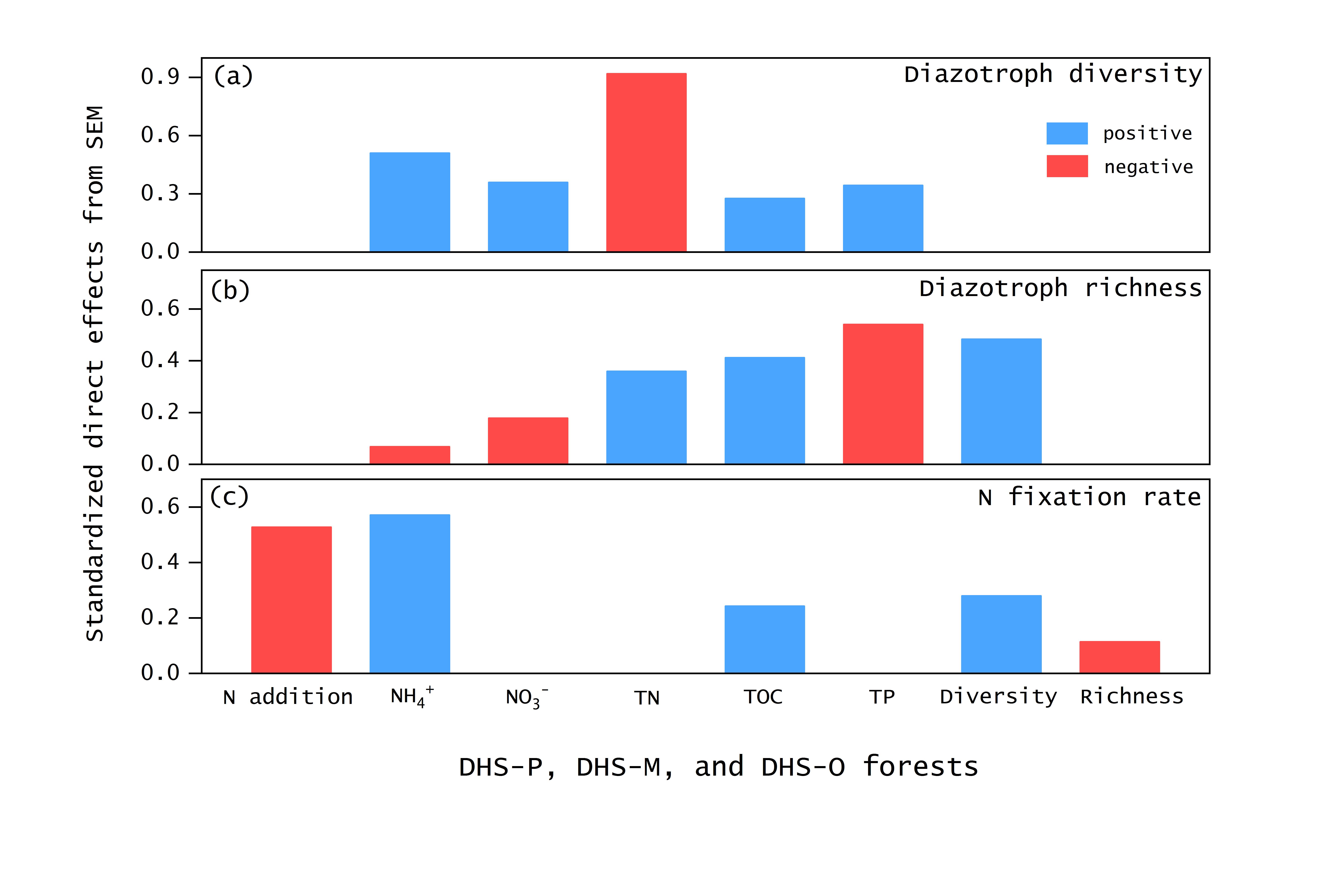


**Fig. S8** Standardized direct effects of nitrogen (N) addition, carbon (C) and nutrient concentrations on diazotroph richness **(a)**, diversity **(b)**, and N fixation rates **(c)** in the Dinghushan pine (DHS-P), mixed (DHS-M), and old-growth (DHS-O) forest soils from structure equation models (SEM). Blue and red colors represent positive and negative effects, respectively. TOC: total organic carbon; TN: total nitrogen; TP: total phosphorus; NO_3_^-^: nitrate; NH_4_^+^: ammonium.


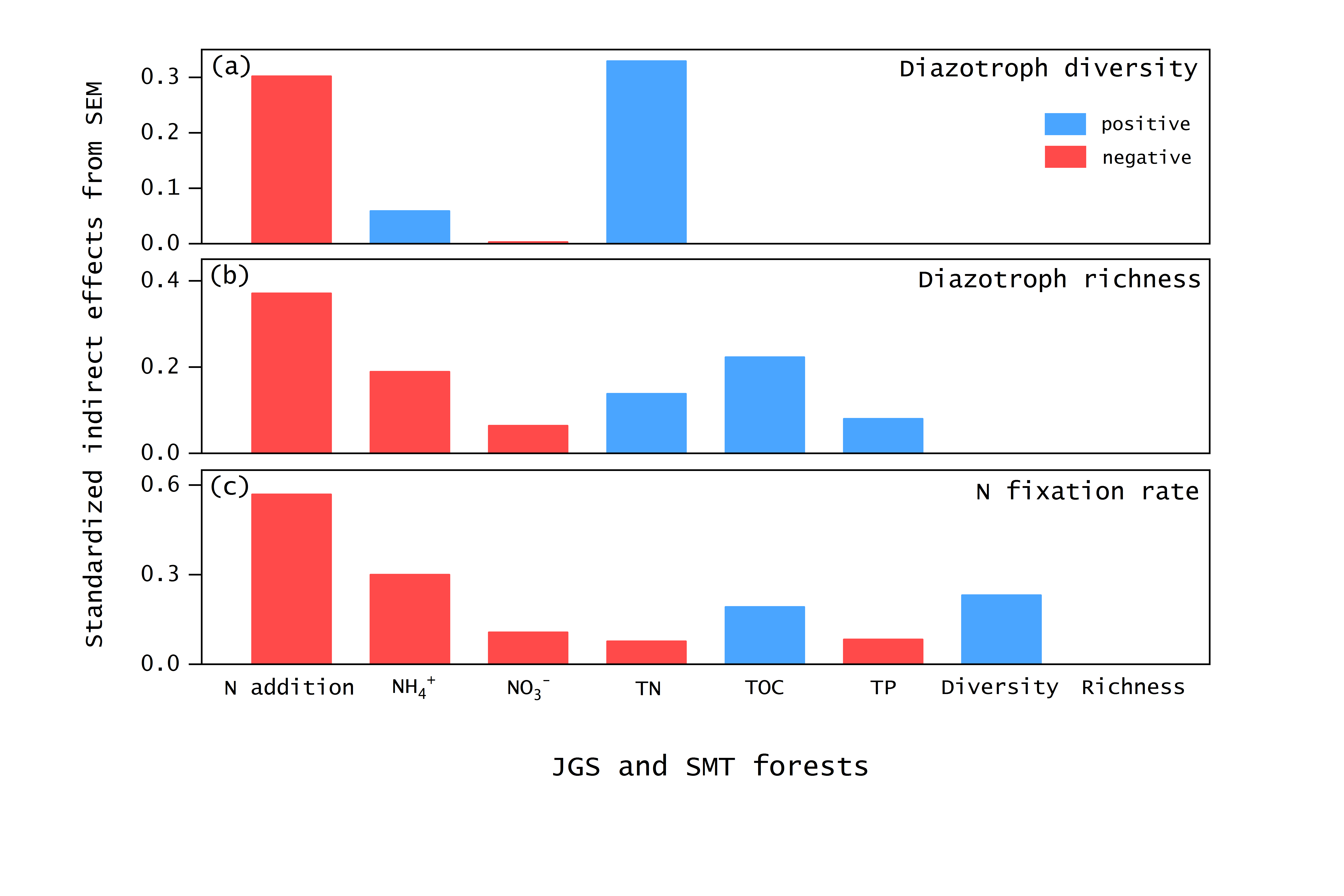


**Fig. S9** Standardized indirect effects of nitrogen (N) addition, carbon (C) and nutrient concentrations on diazotroph richness **(a)**, diversity **(b)**, and N fixation rates **(c)** in the Jigongshan (JGS) and Shimentai (SMT) forest soils from structure equation models (SEM). Blue and red colors represent positive and negative effects, respectively. TOC: total organic carbon; TN: total nitrogen; TP: total phosphorus; NO_3_^-^: nitrate; NH_4_^+^: ammonium.


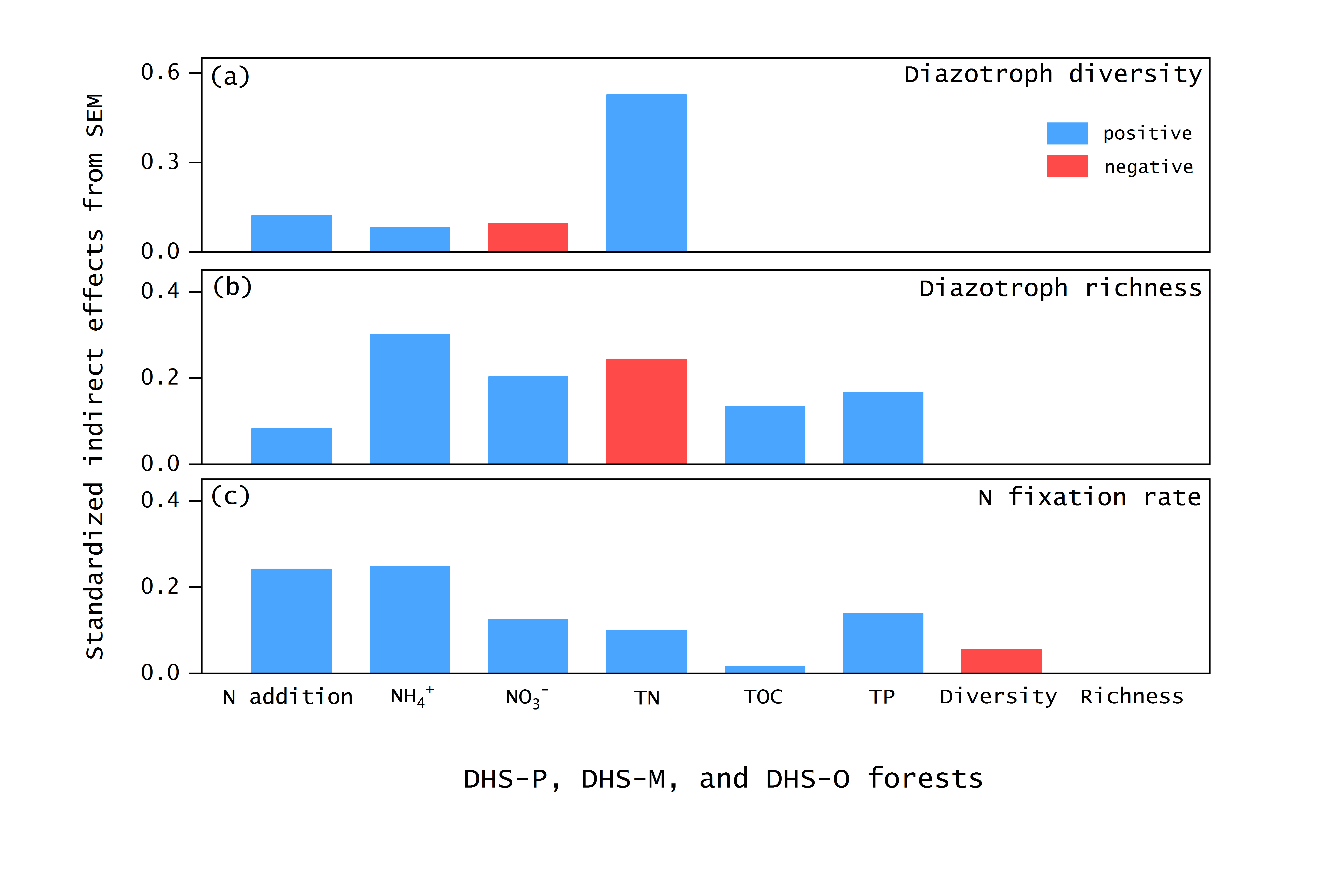


**Fig. S10** Standardized indirect effects of nitrogen (N) addition, carbon (C) and nutrient concentrations on diazotroph richness **(a)**, diversity **(b)**, and N fixation rates **(c)** in the Dinghushan pine (DHS-P), mixed (DHS-M), and old-growth (DHS-O) forest soils from structure equation models (SEM). Blue and red colors represent positive and negative effects, respectively. TOC: total organic carbon; TN: total nitrogen; TP: total phosphorus; NO_3_^-^: nitrate; NH_4_^+^: ammonium.

**Table S1** Soil physicochemical properties of five forest sites following nitrogen (N) addition treatments.

| Forest type | Treatment | TOC (g/kg) | TN (g/kg) | TP (g/kg) | NH_4_^+^ (mg/kg) | NO_3_^-^ (mg/kg) |
| --- | --- | --- | --- | --- | --- | --- |
| JGS | C | 59.55 ± 10.77 | 1.79 ± 0.17 | 0.36 ± 0.14 | 6.42 ± 2.08 b | 4.09 ± 1.82 |
|  | CN25 | 58.37 ± 8.89 | 1.90 ± 0.21 | 0.23 ± 0.04 | 7.14 ± 0.34 b | 6.93 ± 2.25 |
|  | UN25 | 79.66 ± 13.63 | 2.43 ± 0.38 | 0.29 ± 0.01 | 14.15 ± 1.60 a | 5.37 ± 1.06 |
|  | CN50 | 76.85 ± 6.75 | 2.30 ± 0.26 | 0.32 ± 0.08 | 7.34 ± 2.79 b | 8.98 ± 1.57 |
|  | UN50 | 8.40 ± 5.86 | 3.24 ± 0.97 | 0.45 ± 0.12 | 16.30 ± 2.18 a | 6.52 ± 1.32 |
| SMT | C | 25.35 ± 4.25 | 1.95 ± 0.19 | 0.35 ± 0.05 | 8.13 ± 1.14 b | 7.56 ± 1.85 |
|  | CN25 | 32.80 ± 3.40 | 1.62 ± 0.19 | 0.36 ± 0.12 | 11.18 ± 1.33 ab | 9.95 ± 1.62 |
|  | UN25 | 22.64 ± 5.14 | 2.16 ± 0.30 | 0.37 ± 0.12 | 14.06 ± 1.97 a | 11.49 ± 2.01 |
|  | CN50 | 32.74 ± 6.60 | 2.18 ± 0.28 | 0.40 ± 0.14 | 14.98 ± 1.44 a | 9.93 ± 0.60 |
|  | UN50 | 29.51 ± 3.52 | 2.28 ± 0.27 | 0.46 ± 0.08 | 13.91 ± 2.23 a | 10.94 ± 1.51 |
| DHS-P | C | 26.80 ± 1.71 | 1.52 ± 0.07 | 0.26 ± 0.01 | 2.01 ± 0.50 | 4.21 ± 0.56 |
|  | N150 | 22.42 ± 1.02 | 1.61 ± 0.08 | 0.23 ± 0.02 | 3.53 ± 0.44 | 5.56 ± 0.60 |
| DHS-M | C | 34.71 ± 2.40 | 2.07 ± 0.10 | 0.29 ± 0.01 | 2.58 ± 0.63 | 3.68 ± 0.49 |
|  | N150 | 37.39 ± 2.29 | 2.35 ± 0.11 | 0.32 ± 0.02 | 2.87 ± 0.37 | 4.39 ± 0.55 |
| DHS-O | C | 41.19 ± 1.75 | 2.81 ± 0.08 b | 0.38 ± 0.02 | 3.78 ± 0.73 | 5.92 ± 0.34 b |
|  | N150 | 42.95 ± 2.41 | 3.14 ± 0.08 a | 0.35 ± 0.02 | 5.92 ± 0.85 | 7.46 ± 0.49 a |

Note: Values are means ± standard errors. Different lowercase represents statistical significance (*p*<0.05) between the control and N-addition plots. C: control; CN25 and UN25: canopy and understory N addition at the rate of 25 kg N ha^-1^ yr^-1^, respectively; CN50 and UN50: canopy and understory N addition at the rate of 50 kg N ha^-1^ yr^-1^, respectively; N150: understory N addition at the rate of 150 kg N ha^-1^ yr^-1^. JGS: Jigongshan forest; SMT: Shimentai forest; DHS-P: Dinghushan pine forest; DHS-M: Dinghushan mixed forest; DHS-O: Dinghushan old-growth forest.

**Table S2** Soil pH of five forest sites following nitrogen (N) addition treatments.

|  | **C** | **CN25** | **UN25** | **CN50** | **UN50** | **N150** |
| --- | --- | --- | --- | --- | --- | --- |
| **JGS** | 4.36 ± 0.09 | 4.04 ± 0.04 ***** | 4.13 ± 0.02 | 4.14 ± 0.06 | 4.13 ± 0.05 | - |
| **SMT** | 3.95 ± 0.05 | 3.90 ± 0.05 | 3.96 ± 0.06 | 3.86 ± 0.04 | 3.73 ± 0.07 | - |
| **DHS-P** | 4.04 ± 0.03 | - | - | - | - | 3.90 ± 0.07 |
| **DHS-M** | 4.00 ± 0.05 | - | - | - | - | 3.92 ± 0.05 |
| **DHS-O** | 3.86 ± 0.05 | - | - | - | - | 3.85 ± 0.04 |

Note: Values are means ± standard errors. ‘*’ represents statistical significance (*p*<0.05) between the control and N-addition plots. C: control; CN25 and UN25: canopy and understory N addition at the rate of 25 kg N ha^-1^ yr^-1^, respectively; CN50 and UN50: canopy and understory N addition at the rate of 50 kg N ha^-1^ yr^-1^, respectively; N150: understory N addition at the rate of 150 kg N ha^-1^ yr^-1^. JGS: Jigongshan forest; SMT: Shimentai forest; DHS-P: Dinghushan pine forest; DHS-M: Dinghushan mixed forest; DHS-O: Dinghushan old-growth forest.
